# Influence of Harvesting Stages on Phytonutrients and Antioxidant Properties of Leaves of Five Purple-Fleshed Sweet Potato (*Ipomoea batatas*) Genotypes

**DOI:** 10.3390/foods13111640

**Published:** 2024-05-24

**Authors:** Lavhelani Tshilongo, Sephora Mutombo Mianda, Faith Seke, Sunette M. Laurie, Dharini Sivakumar

**Affiliations:** 1 Department of Crop Sciences, Tshwane University of Technology, Pretoria 0183, South Africa; lavhelanit@gmail.com (L.T.); sivakumard@tut.ac.za (D.S.); 2 Agricultural Research Council—Vegetable, Industrial and Medicinal Plants, Pretoria 0001, South Africa; 3 Centre for Nutrition and Food Sciences, Queensland Alliance for Agriculture and Food Innovation, Indooroopilly, QLD 4068, Australia

**Keywords:** morphological traits, caffeoylquinic acid derivative, cyanidin glycosides, peonidin glycosides, carotenoids, ferric reducing antioxidant power, mineral analysis

## Abstract

Sweet potatoes (*Ipomoea batatas*) are highly profitable, contribute to food security, and their leaves rich in phytonutrients. This study examined the optimal leaf harvesting stage by harvesting newly formed leaves (leaves 1 to 5) to achieve the highest concentration of carotenoids, phenolic compounds, antioxidant properties and mineral content. Leaves of five purple-fleshed sweet potato genotypes ‘2019-11-2’ and ‘2019-1-1’, ‘Purple-purple’, and from the USA ‘08-21P’ and ‘16-283P’ were harvested based on tuber life cycle [vegetative 8 weeks after planting (VS-8WAP), tuber initiation (TIS-12WAP), and tuber maturation phases (TMS-16WAP)]. At the 8WAP stage, leaves of genotype ‘2019-11-2’ had the highest concentrations of cyanidin-caffeoyl-sophoroside-glucoside (17.64 mg/kg), cyanidin-caffeoyl-feruloyl-sophoroside-glucoside (41.51 mg/kg), peonidin-caffeoyl-hydroxybenzoyl-sophoriside-glucoside (45.25 mg/kg), and peonidin caffeoyl-feruloyl-sophoriside-glucoside (24.47 mg/kg), as well as antioxidant scavenging activity. In contrast, ‘Purple-purple’ harvested at TIS-12WAP showed the highest concentration of caffeoylquinic acid derivatives. Zeaxanthin, lutein, all trans-β-carotene, and cis-β-carotene are the most abundant carotenoids in genotype ‘08-21P’ at VS-8WAP. As a result, local genotypes ‘2019-11-2’ harvested at 8WAP and ‘Purple-purple’ harvested at 12WAP are potential sources of anthocyanins and caffeoylquinic acid derivatives. Conversely, USA’s genotype ‘08-21P’ at the VS-8WAP stage is an excellent source of carotenoids. The leaves of USA’s ‘08-21P’ genotype and the local ‘2019-11-2’ genotype at TMS-16WAP exhibited the highest content of Fe and Mn, respectively. The study identified the optimal leaf stage for consumption of leaves and for use as a functional ingredient.

## 1. Introduction

Sweet potato (*Ipomoea batatas*), belonging to the family Convolvulaceae, could be a valuable food for coping with future changes in food supply and demand in developing countries [1]. Apart from tubers, sweet potato leaves (SPLs) have the potential to serve as a natural dietary source with the possibility of being developed further as a sustainable crop for multiple uses in the food and medicinal industries [2]. Leafy vegetable crops are an affordable source of bioactive compounds and antioxidants that can potentially combat hidden hunger in rural communities. These antioxidants prevent or inhibit other molecules during oxidation [3]. Research investigations are focusing on identifying fruits and vegetables that are rich in essential phytochemicals and have nutritional benefits in preventing diseases and promoting good health by maintaining a healthy and balanced diet [4]. One such vegetable that is gaining popularity in developing countries is SPLs. In Taiwan, purple SPLs are widely grown as a healthy vegetable [5]. They are a leafy, edible vegetable that is nutritious and readily available throughout the season due to their dual-purpose cropping approach. Spinach, kale, mustard, lettuce, and traditional leafy vegetables like Amaranthus, spider plant, black nightshade, cucurbits, and cowpea leaves are among the most consumed worldwide [6].

Recently, there has been a growing interest in purple-fleshed sweet potatoes because they are rich in anthocyanins. Anthocyanins are a type of phenolic compound that offer many health benefits, and they can be found in the flesh, skin, and leaves of sweet potatoes [2,7]. Researchers have identified 15 different types of anthocyanins in sweet potatoes, including peonidin 3-caffeoyl-p-coumaryl sophoroside-5-glucoside and a pelargonidin type, which were recently discovered [8,9]. Interestingly, the number of anthocyanins in sweet potato cultivars varies, which is why some parts of the plant have a purple pigmentation. Although there is no official RDA for anthocyanins, China recommends 50 mg/day [10]. SPLs are an excellent source of essential vitamins, minerals, and carotenoids, which are crucial for human health. According to studies conducted by Nguyen et al. [2] and Zhang et al. [11], SPLs have higher levels of these compounds compared to other leafy vegetables and even the roots of the plant. Additionally, SPLs can be harvested multiple times, until prior to the harvesting of tubers, making them a competitive option for leafy vegetable crops, as noted by Chirwa-Moonga et al. [12]. Furthermore, SPL has a higher polyphenol content compared to spinach [13]. The SPLs are available and ready to be harvested throughout the growing season, at multiple times within short intervals, as short as weekly harvesting [14]. But harvesting too frequently may reduce the leaf’s nutritional content and retard tuber growth when grown for dual purposes [15]. Gakige et al. stated that the high total biomass could be obtained by harvesting SPL partially after 75 days [16]. Therefore, the harvesting interval plays a role in the yield of harvestable leaves. Furthermore, SPL must be harvested along with young apical buds to supplement nutrients in addition to those of leaves [17]. This is ideal because the apical buds showed the highest level of TPC and a more potent antioxidant activities (AA) than young tender leaves [18]. In recent years, sweet potato breeding has focused on developing cultivars with nutritional benefits, high yield, long storability, and resistance to pests and diseases. While the leaves are identified as suitable for consumption. Purple-fleshed sweet potatoes are being introduced locally and might as well be promoted for dual-purpose use. Developing a niche market for the leaves of these genotypes is ideal as it will improve the cultivation of purple-fleshed sweet potato tubers, thus increasing the overall utilization of sweet potatoes. Currently, the Agriculture Research Council in South Africa has new breeding lines of purple genotypes as well as imported lines under trial for possible commercialisation, and there is little information available on the phytonutritional properties of these purple-fleshed genotypes. Phenolic contents differ with the harvesting stages but to fully understand this trend, the translocation of nutrients to which storage sink (organ) during the stage at which leaves were harvested must be taken into consideration.

In order to achieve the highest concentration of carotenoids, phenolic compounds, antioxidants, and minerals in five purple-fleshed sweet potato genotypes, including ‘2019-11-2’, ‘2019-1-1’, ‘Purple-purple’, and USA’s ‘08-21P’ and ‘16-283P’, this study examined the optimal harvesting stage by harvesting newly formed leaves (leaves 1 to 5) based on tuber life cycle phases [vegetative 8 weeks after planting (VS-8WAP), tuber initiation (TIS-12WAP), and maturation (TMS-16WAP). The results obtained from this study would be beneficial to sweet potato farmers.

## 2. Materials and Methods

### 2.1. Chemicals

The chemicals used for carrying out the experimental procedures were purchased from Sigma Aldrich from Johannesburg, South Africa. Chemicals purchased included 2,2-diphenyl-2-picrylhydrazyl (DPPH, purity 97%, Sigma Aldrich), 2,2′-azino-bis(3-ethylbenzothiazoline-6-sulfonic acid) diammonium sulphate (ABTS, purity ≥ 98%, Sigma Aldrich), 2,4,6-Tris(2-pyridyl)-s-triazine (TPTZ, purity ≥ 99%, Sigma Aldrich), (±)-6-hydroxy-2,5,7,8-tetramethylchroman-2-carboxylic acid (TROLOX, purity 97%, Sigma Aldrich), chlorogenic acid (HPLC, purity 91.5%, HWI pharma services GmbH, Ruelzheim, Germany), rutin (HPLC, purity ≥ 94%, Sigma Aldrich CHEMIE Gmbh, Steinheim, Germany), zeaxanthin (HPLC, purity ≥ 85%, Shanghai MacLean Biochemical Technology Co., Ltd, MACKLIN^®^, Shanghai, China), β-carotene (HPLC, Merck, Rahway, NJ, USA), Lutein (HPLC, purity ≥ 90%, Shanghai MacLean Biochemical Technology Co., Ltd., MACKLIN^®^, Shanghai, China), and Folin–Ciocalteu reagent (Radchem (PTY) LTD, Alberton, Gauteng, South Africa). All HPLC solvents (methanol, acetonitrile, methyl-tert-butyl ether) were purchased from Sigma Aldrich, Johannesburg, South Africa. Deionized water was purchased from Glassworld, Johannesburg, South Africa.

### 2.2. Experimental Site

The trial was planted at the Agricultural Research Council for Vegetable, Industrial and Medicinal Plants known as ARC-VIMP located along Moloto/Kwa Mhlanga Road in Roodeplaat outside Pretoria in Gauteng (GPS coordinates: 25′56° S; 28′35° E), 1164 m above sea level [19]. According to Köppen classification, the area is categorised as a humid subtropical (Cwa) region due to its warm temperate climate with a clay loam type of soil [20]. Climatic data were collected by the WT1080 professional weather station throughout each growing season. External environmental factors such as temperature (minimum and maximum), rainfall, and humidity (%) from January to May in 2022 (Season 1, S1) and 2023 (Season 2, S2) were recorded during the growth period (Figure S2A,B of the Supplementary File). The PAR (µmol/m^2^s) on the day of harvest was also recorded using an ACCUPAR LP-80 (PAR/LAI) ceptometer (Decagon devices, Washington, DC, USA).

### 2.3. Cultivation

Five purple-fleshed genotypes were used as treatments, including ‘Purple-purple’ (local), ‘08-21P’, and ‘16-283P’ imported from the USA, as well as two ARC breeding lines, ‘2019-11-2’ and ‘2019-1-1’ (Figure S1 of Supplementary File). Cuttings that were approximately 30 cm long with 3 or 4 nodes were taken from mature vines and prepared before planting. Fertilisation and field preparation were conducted in accordance with the ARC guidelines for sweet potato production in South Africa [21]. The field was prepared with ridges 1 m spacing apart and 0.3–0.4 m high. Plants were spaced at 0.3 m between the plants on the ridge [19]. The 2022 trial was planted at land no. 41 N with a total of five blocks each with a population of 20 plants per treatment (5 genotypes) without replications. In 2023, after the generation advanced in the breeding scheme, the second trial was planted in land no. 30, which is adjacent to land no. 41. Treatments (genotypes) were replicated in triplicate in a randomised complete block design with each block consisting of 20 plants per replicate.

Table S1 of Supplementary File shows the properties and nutritional status of the soil in plot no. 41 and plot no. 30. Based on the soil analysis and fertiliser recommendation report, 500 kg/ha of 1:0:1 (37%) plus 200 kg/ha of superphosphate (12%) of fertiliser was applied at planting. After the planting of the sweet potato cuttings, the top dressing was applied as 150 kg/ha of LAN (28). Twenty-one days after planting, a further 150 kg of LAN (28) plus 250 kg of potassium chloride per hectare were applied as top dressing. Irrigation was scheduled twice a week during the first six weeks after planting and then reduced to once a week after establishment. Irrigation was applied for 2 h with sprinkler irrigation supplying approximately 25–30 mm of water weekly, depending on the rainfall. To protect the plants from pests, Decis^®^ (Bayer CropScience, Maharashtra, India) was sprayed, and Cymoxazeb^®^ (Villa Crop Protection, Kempton Park, Gauteng, South Africa) was applied to protect the plants from fungal diseases.

### 2.4. Harvesting of Leaves and Preparation of Samples

**Figure 1:**
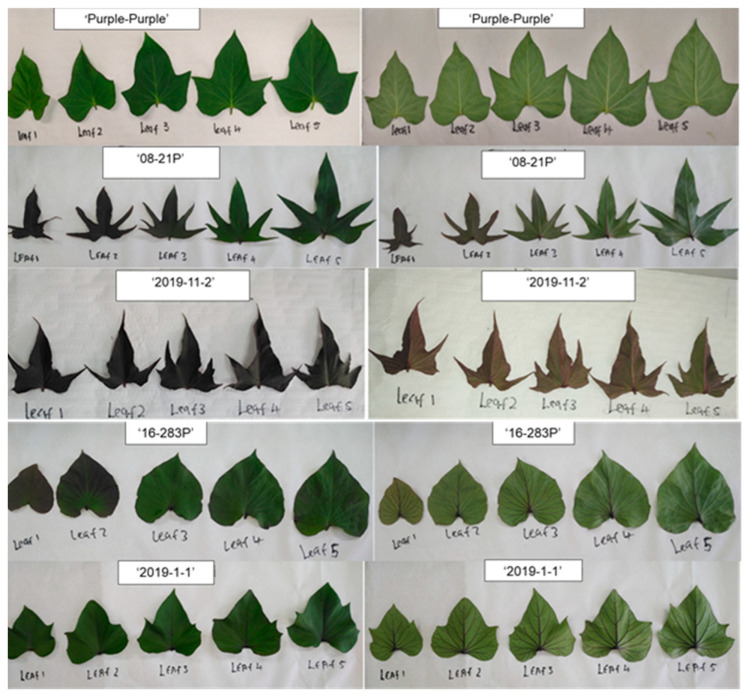
Colour development on the leaf blade of purple-fleshed genotypes within the first five harvestable leaves on vine tips.

The leaves were harvested three times over two growing seasons in 2022 and 2023 from 8 weeks after planting (8WAP) to 16 weeks after planting (16WAP) at four-week intervals. Week 8 occurred during the vegetative stage, week 12 at tuber initiation, and week 16 during the tuber maturation stage of sweet potato plant growth stages. SPLs were harvested by randomly hand-picking a maximum of five newly formed leaves. The leaves were from leaf developmental position 1 to 5 on the vine growth tips with each harvesting stage (Figure 1). Figure 1 also shows the colour development on the leaf blades of purple-fleshed genotypes within the first five harvestable leaves on vine tips. A set of 500 g per genotype were harvested and transported at 15 °C to the laboratory. Leaves were then sorted by removing petioles, discarding damaged leaves, and washed with running water to remove soil. After freezing at −80 °C, the leaf blades were freeze dried using a SP scientific virtis benchtop pro freeze dryer (BTP-9SGE0X, NY, USA) at −55 °C, at a total pressure of 2 × 10 + 3 mbar, for 7 days then ground into powder. The powders were used for all biochemical analysis.

### 2.5. Analysis

#### 2.5.1. Morphological Analysis

The sweet potato’s morphological characteristics were identified using international descriptors as outlined by Huamán [22]. Several phenotypic traits, such as leaf shape, number of lobes, and abaxial vein pigmentation were recorded, along with the mature and immature leaf colours.

#### 2.5.2. Colour and Chlorophyll

CR-400 chroma meter (Konica Minolta Sensing, Osaka, Japan) was used to measure the colour. A white plate was used to calibrate the Chroma meter before use [23]. In each genotype, 10 readings were taken from different locations on the abaxial and adaxial leaf surfaces. Three sets of data were averaged and used as a representative sample. The colour value was recorded using the L*, a*, and b*. L* represents luminosity ranging from 0 to 100 (the highest value means the lighter the leaves); a* value ranges from −128 to 127 representing greenness to redness; and b* represents yellowness to blueness with values ranging from −128 to 127 [24].

A portable soil plant analysis development (SPAD) chlorophyll meter (SPAD-502 plus, Konica Minolta Sensing, Osaka, Japan) determined the chlorophyll content in SPLs non-destructively by recording the greenness from various leaf points. Before use, the SPAD meter was calibrated by clamping the measuring head without a sample. The measurements were replicated three times taking 10 repeated measures, and averages were used as a representative of three sets of data. The results were recorded in μg/cm^2^ units.

#### 2.5.3. Leaf Area Index (LAI)

The LAI was measured using the ACCUPAR LP-80 (PAR/LAI) ceptometer (Decagon devices, Washington, DC, USA). Ten repetitive measures were taken above and below the leaf surface with three replicates to obtain an LAI.

#### 2.5.4. Mineral Analysis

The leaf samples were tested at ARC-Vegetable, Industrial and Medicinal Plants analytical facility in Rustenburg (ARC-VIMP). Micro Kjeldahl acid digestion process was used for N, P, and K 2 and dry-ashing and further digestion with Aqua Regia for Ca, Mg, and Micronutrient.

#### 2.5.5. Extraction for Total Phenol Content (TPC) and Antioxidant Activities Assays

Ten mg of freeze-dried samples were weighed and mixed thoroughly with 2 mL of 80% methanol using a vortex mixer (BV1000, Benchmark Scientific Inc., New Jersey, USA) for 60 s, then sonicated for 10 min at 35 °C in a DC-150H MRC ultrasonic cleaner [23]. After that, the sample was centrifuged for 20 min at 4427× *g* with a Hermle centrifuge (Z326k, Hermle Labortechnik GmbH, Wehingen, Germany). Extraction was repeated three times, and the resulting supernatants were pooled together into 1. Analysis of TPC, FRAP, ABTS, and DPPH was carried out using the collected supernatants.

#### 2.5.6. TPC

The TPC was carried out following the method described by Phahlane et al. [25] using Folin-Cioclateu reagent with minor changes. A total of 0.5 mL of diluted extract (100 μg/mL) was mixed together with 2.5 mL of Folin–Ciocalteu reagent (diluted 10 times with water), and 2 mL of Na_2_CO_3_ (75 g/L). The mixture was allowed to stand for 2 h at room temperature and absorbance was measured through a UV-Vis Spectrophotometer (SPECTROstar^®^Nano BMG Labtech, Ortenberg, Germany) at 765 nm. The results were expressed as chlorogenic acid equivalent (CAE) mg/g Dw (Dry weight of leaf extract) based on the calibration curve (Y = 0.0034x + 0.0569, µg/mL, R^2^ = 0.99, linear range: 0–100 μg/mL). Chlorogenic acid is the major phenolic acid in sweet potatoes.

#### 2.5.7. Ferric Reducing Antioxidant Power (FRAP)

FRAP value was determined as described by Phahlane et al. [25] without any changes. The FRAP reagent was prepared by mixing acetic acid buffer pH 3.6, TPTZ solution (10 mmol/L), and FeCl_3_ (20 mmol/L) solution according to the ratio (10:1:1). Then the mixture was incubated at 37 °C. Within 1–2 h, 240 µL of FRAP reagent was mixed with 10 µL of leaf extract (Section 2.5.5) into several wells on a microplate reader. The mixture was left a room temperature in darkness to react for 5 min before spectrophotometer (BMG LABTECH GmbH, Spectro Star Nano, Ortenberg, Germany) readings, measuring the absorbance at 593 nm. The results were calculated against the calibration curve Y = 0.0004x + 0.0825 (µg/mL, R^2^ = 0.97, linear range: 0–100 µmol/mL) of the TROLOX standard and expressed as TEAC mM/g dw (dry weight), which was calculated as follows:

$$FRAP\;value\;(\mu mol\;TEAC/g\;dw)=\frac{c\times V\times DF}{m}$$

where c is the sample concentration (µmol/mL) calculated against the TROLOX standard curve, V is the volume of the sample (mL), DF is the dilution factor, and m is the weight of the sample dry matter (g).

#### 2.5.8. 2,2-Azino-bis-3-ethylbenzothiazoline-6-sulfonic (ABTS) Scavenging Activity

SPL scavenging activity with ABTS assay was carried out according to the procedure described by Phahlane et al. [25]. Prior to use, 5 mL of ABTS stock solution was added to 45 mL of phosphate buffer. Serial dilutions from the sample extract (Section 2.5.5) were made and transferred into wells in a microplate reader and thereafter incubated for 30 min in the dark to allow a reaction with 200 µL of ABTS stock solution. The absorbance readings were obtained at 750 nm in a spectrophotometer and the results were recorded as IC_50_ mg/mL Dw. IC_50_ is the concentration of extracts required to scavenge 50% of the free radicals where a lower value shows higher radical scavenging efficiency [26]. The formula used to calculate the % inhibition is = (A0 − A1) × 100/A0

whereby A0 and A1 represent the ABTS assay absorbance and samples absorbance, respectively. The % inhibitions (y) were plotted against the sample concentrations (x) at all points, and the respective regression line (y = ax + b) was drawn. The IC_50_ value was calculated by substituting the value of Y with 50 in the regression equation.

#### 2.5.9. 2,2-Diphenyl-1-picrylhydrazyl (DPPH) Radical Scavenging Activity

A DPPH assay was carried out using a DPPH solution according to Phahlane et al. [25]. Briefly, 1.03 mg of DPPH was weighed and dissolved in 31.25 mL of absolute methanol to prepare the DPPH solution. An additional 15.625 mL of methanol diluted the DPPH solution to give the initial absorbance of 0.493 at 517 nm in a spectrophotometer. Thereafter, different concentrations (0–10 mg/mL) from the sample extract (Section 2.5.5) were made and 100 µL of each were transferred into a microplate reader wells (BMG LABTECH GmbH, Spectro Star Nano, Ortenberg, Germany) and incubated in darkness to allow a reaction with 200 µL of DPPH for 30 min before reading the absorbance. The formula used to calculate the % inhibition is = (A0 − A1) × 100/A0, whereby A0 and A1 represent the DPPH absorbance and sample absorbance, respectively [27]. The % inhibitions (y) were plotted against the sample concentrations (x) at all points, and the respective regression line (y = ax + b) was drawn. The IC_50_ value was calculated by substituting the value of Y with 50 in the regression equation.

#### 2.5.10. Total Carotenoids

Total carotenoids were measured following the process performed by Phahlane et al. [25]. For carotenoids extraction, 100 mg of freeze-dried sweet potato leaves were homogenised with 5 mL of 95% ethanol containing 0.1% (*w*/*v*) butylated hydroxytoluene (BHT) for 10 min. The mixture was saponified using KOH solution (20% in methanol, *w*/*v*) for 30 min under constant agitation. Carotenoids were then extracted three times with 3 mL of a mixture of (70:30, *v*/*v*) hexane/dichloromethane containing 0.1% BHT. A solution of 10% NaCl (*w*/*v*) was used for phase separation and then the mixture was centrifuged at 3900× *g* for 5 min. The upper layer was collected, combined, and the solvent was evaporated under nitrogen stream to dryness. A total of 2 mL of methanol/MTBE (50:50, *v*/*v*) prepared with 0.1% BHT was used to redissolve the carotenoids after drying under a nitrogen stream. A standard stock solution of β-carotene was prepared by dissolving 1 mg in 10 mL of methanol/MTBE. Standard solutions with concentrations of 0 to 0.1 mg/mL were prepared from serial dilution of stock solution. The absorbance of reconstituted extracts and standards were read at 460 nm using a spectrophotometer and the results were calculated against the β-carotene standard calibration curve Y = 0.0206x + 0.0318 (R^2^ = 0.99, linear range: 0–100 µg/mL) on a dry weight basis in mg/g.

#### 2.5.11. Quantification of Phenolic and Anthocyanin Compounds Using UPLC-QTOF/MS

A Waters Cyclic Quadrupole time-of-flight (qTOF) mass spectrometer (MS) connected to a Waters Acquity ultra-performance liquid chromatograph (UPLC) (Waters, Milford, MA, USA) was used for high-resolution UPLC-MS analysis. Column eluate first passed through a Photodiode Array (PDA) detector before going to the mass spectrometer, allowing simultaneous collection of UV and MS spectra. Electrospray ionization was used in positive mode with a cone voltage of 15 V, desolvation temperature of 275 °C, desolvation gas at 650 L/h, and the rest of the MS settings optimized for best resolution and sensitivity. Data were acquired by scanning from *m*/*z* 100 to 1500 *m*/*z* in resolution mode as well as in MSE mode. In MSE mode two channels of MS data were acquired, one at a low collision energy (4 V) and the second using a collision energy ramp (40–100 V) to obtain fragmentation data as well. Leucine enkaphalin was used as lock mass (reference mass) for accurate mass determination and the instrument was calibrated with sodium formate. Separation was achieved on a Waters HSS T3, 2.1 × 150 mm, 1.7 μm column. An injection volume of 0.5 μL was used and the mobile phase consisted of 0.1% formic acid (solvent A) and acetonitrile containing 0.1% formic acid as solvent B. The gradient started at 100% solvent A for 1 min and changed to 28% B over 22 min in a linear way. It then went to 40% B over 50 s and a wash step of 1.5 min at 100% B, followed by re-equilibration to initial conditions for 4 min. The flow rate was 0.3 mL/min, and the column temperature was maintained at 60 °C. Compounds were quantified in a relative manner against a calibration curve established by injecting a range of neochlorogenic acid and rutin standards from 0.2 to 5 mg/L rutin. After compression, centroiding and application of lock mass correction, the data were processed using MSDIAL and MSFINDER (RIKEN Center for Sustainable Resource Science: Metabolome Informatics Research Team, Kanagawa, Japan) [28,29]. Databases searched by MSFinder: Functions 1 (unfragmented channel) and 2 (fragmented channel) of the Waters MSe data were processed by MSDial to produce MS1 and MS2 spectra as well as extracted ion chromatograms with associated peak height intensity data. Since calibration standards were not available for the majority of these compounds, the peak height intensity was converted to concentration in a semi-quantitative manner by interpolation off a calibration curve for neochlorogenic acid acquired under the same instrumental conditions.

Each deconvoluted feature (alignment in MSDial), together with its associated MS1 and MS spectra was exported from MSDial to MSFinder. Based on the accurate mass elemental compositions, possible compounds were identified from the listed databases and then subjected to in silico fragmentation. According to the spectral match between the in silico and measured spectra, a score (out of 10) is assigned to each of the possible compound matches with the highest score being accepted as the most likely (assuming a score of at least 4). The content of phenolic and anthocyanin compounds in the study is expressed as mg/kg.

#### 2.5.12. Quantification of Carotenoid Components Using HPLC-PDA

After drying under a nitrogen stream, sample extracts were reconstituted in 0.6 mL of methanol/MTBE (50:50 *v*/*v*) prepared with 0.1% BHT and injected (10 µL) into an HPLC (Shimadzu Prominence-i HPLC System with DAD Detector, Shimadzu, Kyoto, Japan) system [25]. The chromatographic separation was achieved on a YCM C30 column (3.6 × 250 mm, 3.6 µm) (Waters, Milford, MA, USA) kept at a consistent temperature (25 °C), with eluent A and B (solutions of 0.1% formic acid in methanol and 0.1% formic acid in methyl tert-butyl ether, respectively). The linear gradient was 0 min 0% B, 1 min 5% B, 37 min 50% B, and 38 min 0% B. Pure external standards (zeaxanthin, cis-β-carotene, lutein, all trans-β-carotene, and zeaxanthin) were used to identify and quantify carotenoids in the sample extract. The chromatograms were recorded at 460 nm. The calibration curves were obtained using different concentrations of the standards (0–0.1 mg/mL). Each carotenoid is presented as mg/100 g dry weight.

### 2.6. Statistical Analysis

The leaves were harvested three times in one season over two years. To see the significant difference between the harvesting stage and genotypes, a set of three data values was analysed statistically with two-way ANOVA using GenStat (64-bit Release 22.1, PC windows 10, VSN International Ltd., Hertfordshire, UK) and the results were presented as mean and standard deviation (*n* = 3). Tukey’s HSD was adopted to differentiate the means between the treatments at a 5% significance level.

## 3. Results and Discussion

### 3.1. Morphological Characterisation

Table S2 of Supplementary File shows the variation in leaves among five purple-fleshed sweet potato genotypes. The evaluation of leaf outline, leaf lobe type, leaf lobe number, and abaxial leaf vein was conducted to determine the key quality traits that explain the variation between sweet potato cultivars [19]. Among the five genotypes, ‘08-21P’ and ‘2019-11-2’ have the same leaf outline but differ in leaf lobe type and number. The genotype ‘08-21P’ can be distinguished from ‘2019-11-2’ based on the number of young purple leaves (1 up to 3) in the vine tip (Figure 1). The occurrence of purple leaves in the vine tips is referred to as a red fading process that protects young leaves and prevents the limitation of photosynthetic capacity at a later stage [30]. The genotypes ‘Purple-purple’, ‘16-283P’, and ‘2019-1-1’ had green leaves from leaves 1 to 5. Leaf developmental stages 1–3 showed slightly purple edges on genotype ‘Purple-purple’ and visible purple pigmentation on genotype ‘16-283P’s’ leaf blade (Figure 1). Genotype ‘Purple-purple’ had a slightly lobed leaf type and genotype ‘2019-1-1’ had a very slightly lobed leaf type, whereas genotype ‘16-283P’ differed completely from the other genotypes with a coordinate leaf outline. On the abaxial side, only ‘Purple-purple’ did not display purple veins.

### 3.2. Leaf Area Index (LAI)

The analysis of variance on LAI indicated significant effects for the harvesting stage (*p* < 0.001), genotype by harvesting stage (*p* < 0.05), and harvesting stage by season (*p* < 0.001) (Table S3 of Supplementary File). LAI ranged from 0.123 to 2.107 m^2^ m^−2^ in season 1 (S1) and between 0.04 to 2.40 m^2^ m^−2^ in S2 (Table S4 of Supplementary File). Mbayaki and Karuku [31] reported LAI ranging from 0.5 to 0.7 (cm^2^ cm^−2^) and 1.1 to 2.2 (cm^2^ cm^−2^) during S1 and S2, respectively. The recommended LAI required to intercept enough sunlight is 3–4 m^2^ [32], and the values obtained in this study were relatively low. Lower values can be related to the genetic composition of varieties. Table S4 of Supplementary File shows genotype ‘16-283P’ x VS-8WAP was significantly (*p* < 0.001) higher than the values obtained in all genotypes in S1 (2.11 m^2^ m^−2^) and S2 (2.41 m^2^ m^−2^). The lowest values were obtained in all genotypes during TMS-16WAP without a significant (*p* > 0.001) difference. Table S4 of Supplementary File shows lower values of LAI in ‘Purple-purple’ x TMS-16WAP (0.17 m^2^ m^−2^), ‘08-21P’ x TMS-16WAP (0.08 m^2^ m^−2^), ‘2019-11-2’ x TMS-16WAP (0.22 m^2^ m^−2^), ‘16-283P’ x TMS-16WAP (0.18 m^2^ m^−2^) and ‘2019-1-1’ x TMS-16WAP (0.18 m^2^ m^−2^) during both seasons. The trend observed with LAI was VS-8WAP > TIS-12WAP > TMS-WAP in all genotypes. TMS-16WAP occurred before the harvesting of tubers, at this stage older leaves are shedding and falling off, hence the low LAI. A similar trend was reported by Mbayaki and Karuku [31] with the LAI being highest during the vegetative stage. Widaryanto and Saitama [32] determined LAI during two, three, and four months after planting, sweet potato cultivars showed variation with each observation period.

### 3.3. Chlorophyll

Year x harvesting stage x genotype significantly (*p* < 0.001) influenced the chlorophyll content (Table S3 of Supplementary File). All other main and interaction effects were also significant except the harvesting stage. Chlorophyll SPAD (soil plant analysis development) readings ranged from 43.47 to 47.66 μg/cm^2^ (Table S3). Koshy et al. [33] reported SPAD readings in sweet potato leaves ranging from 22.1 to 47.8 with an average of 34.76 in 43 sweet potato leaf samples. Genotype ‘2019-1-1’ x TMS-16WAP (47.66 μg/cm^2^) and ‘2019-1-1’ x VS-8WAP had significantly high chlorophyll content. ‘Purple-purple’ x VS-8WAP (43.66), ‘08-21P’ x TMS-16WAP (43.47), and ‘16-283P’ x TMS-16WAP (43.56) had lower chlorophyll content. Chlorophyll content was influenced by the genotype.

### 3.4. Leaf Colour

Colour indicates the presence of primary pigmentation in plants, namely carotenoids (Yellow and orange), anthocyanins (Purple to blue), and chlorophyll (Green) [34]. Table S3 of Supplementary File shows that for L* all main and interaction effects were highly significant (*p* < 0.001). L* represents the luminosity in leaves and the darker the leaves the higher the accumulation of dark pigments (anthocyanins). In Supplementary Table S5A, genotype ‘08-21P’ x VS-12WAP (27.24), ‘08-21P’ x TIS-16WAP (27.05), ‘Purple-purple’ x TMS-16WAP (27.59), ‘2019-1-1’ TIS-12WAP (27.07), ‘16-283P’ X TMS-16WAP (25.51), ‘2019-1-1’ X TIS-12WAP (27.07) were lighter. The darker leaves were obtained in genotype ‘Purple-purple’ x VS-8WAP (17.75), ‘2019-11-2’ x VS-8WAP (18.10), ‘2019-11-2’ x 12WAP (19.34), and ‘16-283P’ x VS-8WAP (18.00). During the leaf harvesting stages, there was a noticeable trend (TMS-16WAP > TIS-12WAP > VS-8WAP) in the L* value on the adaxial side of the leaf. A similar trend can be seen in Figure 1 for the abaxial leaf surface as well as the adaxial leaf surface; ‘08-21P’ x VS-12WAP (37.66), ‘08-21P’ x TIS-16WAP (37.17), and ‘Purple-purple’ x TMS-16WAP (37.21) were lighter. The darker abaxial leaf L* colour was obtained in ‘16-283P’ x VS-8WAP (25.6).

The a* colour coordinate was affected by the genotype, harvesting stage, season, and their interaction (Table S3 of Supplementary File). On both leaf surfaces, genotype ‘2019-11-2’ maintained high values of a* compared to other genotypes. A high a* value indicates a redder colour, and this genotype had purple leaves on the vine tips (Figure 1). During S1, ‘2019-11-2’ x VS-8WAP (−1.943), ‘2019-11-2’ x TIS-12WAP (−1.573), and ‘2019-11-2’ x TMS-16WAP (−1.166) displayed high vales of a* on the adaxial leaf side (Supplementary Table S5B). ‘2019-11-2’ x TIS-12WAP (−0.907) and ‘2019-11-2’ x TMS-16WAP (−0.948) had high a* on the abaxial leaf surface 9. Genotypes ‘08-21P’ x TMS-16WAP (−8.464) and ‘Purple-purple’ (−77.732) had lower values for the adaxial and abaxial leaf surfaces, suggesting the leaves were greener. In season 2, ‘2019-11-2’ x VS-8WAP (-0.033), ‘2019-11-2’ x TIS-12WAP (0.458), and ‘2019-11-2’ x TMS-16WAP (0.449) were redder whereas ‘2019-1-1’ x VS-8WAP (−14.087), ‘2019-1-1’ x TIS-12WAP (−13.559), and ‘Purple-purple’ x TIS-12WAP (−13.721) were greener on the adaxial leaf surface. On the abaxial leaf surface, genotypes ‘2019-11-2’ x VS-8WAP (−1.713), ‘2019-11-2’ x TIS-12WAP (0.082), and ‘2019-11-2’+ x TMS-16WAP (0.679) exhibited high values of a* while ‘Purple-purple’ x TIS-12WAP (−13.353) had low a* value. As opposed to season 1 (2022, 11 to 21 °C), in season 2 (2023) the temperatures were slightly higher (16 to 27.5 °C). During heat-stress conditions, genotypes with lighter leaf colours can reduce heat load by reflecting more solar radiation, resulting in improved leaf function under summer heat conditions [35]. Sweet potato leaves (SPLs) may contain anthocyanin too low for the naked eye [36], negative −a* value indicates the presence of chlorophyll, and positive values suggest the presence of anthocyanins such as pelargonidin, peonidin, and cyanidin with a purple-red-pink colour. In some genotypes that show a slightly higher colour coordinate, leaves turn slightly purple-red under heat stress [37]. Secondary metabolites, such as anthocyanins (red pigments), may play a crucial role in adaptation. Anthocyanins have antioxidant properties that may mitigate the damage caused by reactive oxygen species [37].

All main and interactions were significant with the b* colour value at *p* < 0.001, and genotype x harvesting stage x season influenced the b* value at *p* < 0.01 (Table S3 of Supplementary File). A positive b* value indicates the yellowness of the leaves. The high value indicates yellow leaves, and the low value is less yellow. Leaves of purple leaf genotypes ‘2019-11-2’ x TMS-16WAP (5.55) and ‘2019-11-2’ x TIS-12WAP (6.38) had low b*, while ‘Purple-purple’ x TMS-16WAP (16.65) and ‘2019-1-1’ x TIS-12WAP (16.78) were yellower (supplementary Table S5C; Figure 1). The trend observed follows the L*, TMS-16WAP > TIS-12WAP > VS-8WAP. Leaves harvested during the last harvest (TMS-16WAP) were yellower, this could be due to leaf senescence. On the abaxial leaf surface, genotype x harvesting stage x season did not influence the b* colour value. ‘Purple-purple’ x TMS-16WAP (12.95) and ‘2019-1-1’ X TMS-16WAP (12.36) were yellower in S1 whereas in S1 ‘Purple-purple’ x TIS-12WAP, ‘Purple-purple’ x TMS-16WAP, ‘2019-1-1’ x TIS-12WAP and ‘2019-1-1’ x TMS-16WAP had high b* values. Genotype ‘2019-11-2’ maintained a lower b* throughout the harvesting stages on the abaxial leaf side. This is consistent with the opposite increasing trend shown in Table S5C, probably due to the increasing anthocyanins.

### 3.5. TPC

TPC was significantly influenced by genotype x harvesting stage x season interaction at *p* < 0.001 and all the main effects (Table S6 of Supplementary File). Table 1 shows ‘2019-11-2’ x TIS-12WAP with the highest TPC (262.2 chlorogenic acid equivalent (CAE) mg/100 g dw), and the lowest TPC (85.0 CAE mg/g dw) was obtained in sweet potato leaves (SPLs) harvested at TMS-16WAP in genotype ‘2019-1-1’. The highest leaf TPC was obtained in a purple leaf genotype ‘2019-11-2’ followed by ‘08-21P’, (Table 1), these genotypes have hastate leaf outlines (Figure 1). According to the metabolic profiling by Tan et al. [38], high phenolics were correlated with lobed leaf types, this is consistent with the findings in this present study. Makori et al. [8] and Jia et al. [18] reported high TPC in green arial parts which contradicts the findings of this study. Tan et al. [38] showed a correlation between the leaf colour with flavonoids.

The trend was observed as follows: TIS-12WAP > VS-8WAP > TMS-16WA. SPLs reached their maximum growth peak during the vegetative and tuber initiation stage; thereafter, they ceased to grow and started shedding. The phenolics are secondary metabolites produced in the leaf blades, there is a probability that synthesis of these metabolites reaches the maximum point during these stages, and this could explain the variation in the accumulation of TPC during VS-8WAP and TIS-12WAP. TPC is also induced by temperature [2]. Motsa et al. [4] indicated that favourable environmental conditions allow the plant to store phytochemicals in harvestable parts. On the other hand, elevated temperatures weaken the assimilation process which results in low polyphenol content [39]. This is also evident from our study, the second-season (S2 2023) leaf samples showed lower TPC. On the contrary, Suárez et al. [40] obtained high TPC during the last harvest (H3). This could be explained by plant maturity stage, leaf developmental stage, and environmental conditions. The effect of the harvesting stage on the TPC was reported by Takács-Hájos and Vargas-Rubóczki [39], where beetroot leaves harvested 60 days after planting had higher polyphenols than leaves harvested after 85 days. Krochmal-Marczak et al. [41] reported different concentrations of phenolic compounds in leaves of sweet potato cultivars (Okinawa, Carmen Rubin, Radiosa, White Triumph, Molokai, Purple, Beauregard, Jewel, and Satsumo Imo) grown in Poland, harvested during three different growth stages. The results ranged between 85.0 to 262.2 CAE mg/g dw (Table 1). Sun et al. [42] evaluated 40 different sweet potato cultivars with TPC values ranging between 2730 and 12,460 (equivalent to 27.3–126.4) chlorogenic acid (CHA) mg/100 g dw. Similar findings were reported by Makori et al. [8] in SPLs of four cultivars harvested in Beijing China, the TPC was 7 130-12 080 (equivalent to 71.3–120.8 mg/g dw) CAE mg/100 g dw. Our results are comparably higher, and this could be due to the genotype, harvesting stage, and environmental conditions [41].

### 3.6. UHPLC-QTOF-MS Identification and Characterisation of Phenolic Compounds

Table 1 shows that TPC in S1 > S2. This suggest that high temperatures and light intensity in S2 affected the accumulation of phenolic compounds compared to S1 (Supplementary Figure S2). Anthocyanins, phenolic acids, and flavonoids are phenolic compounds, hence S1 data were presented due to moderate temperatures. Moderate temperatures (20–25 °C) influenced the synthesis of anthocyanins composition [43]. According to Sasaki et al. [44] the content of caffeic acid (CA) and caffeoylquinic acid (CQA) were higher in SPLs grown under 20–25 °C than in warm conditions (30 °C).

#### 3.6.1. Anthocyanins

Anthocyanins in sweet potato leaves are mainly acylated with caffeoyl, p-coumaryl, feruloyl, and p-hydroxybenzoyl [9]. This study detected and quantified mono and deacylated anthocyanins (cyanidin and peonidin derivatives) in leaves of five sweet potato genotypes. The concentration of different anthocyanins in the leaves varies depending on its root’s growth cycle stage and the number of weeks since planting, as shown in Table 2. Five different types of anthocyanin derivatives have been identified in our study, including two cyanidin derivatives (cyanidin-caffeoyl-sophoroside-glucoside and cyanidin-caffeoyl-feruloyl-sophoroside-glucoside) and three peonidin derivatives (peonidin feruloyl-sophoroside-glucoside, peonidin caffeoyl-hydroxybenzoyl-sophoroside-glucoside, peonidin caffeoyl-feruloyl-feruloyl-feruloyl-feruloyl-feruloyl-feruloyl-sophoroside-glucoside). In contrast, Su et al. [9] discovered fourteen anthocyanins in the leaves of three sweet potato genotypes in the USA. Vishnu et al. [45] detected nine acylated anthocyanins in the leaves of purple sweet potato genotypes. Li et al. [46] detected 18 acylated cyanidin and peonidin derivatives anthocyanins in two different cultivars of purple-leaf sweet potatoes, Fushu No. 23, and Fushu No. 317, from China. Meanwhile, Su et al. [9] identified and measured 14 anthocyanins in the leaves of P40, a type of purple-fleshed sweet potato.

Across all samples, the peonidin/cyanidin ratio exceeded 3.5; however, cyanidin-caffeoyl-feruloyl-sophoroside-glucoside, peonidin-caffeoyl-hydroxybenzoyl-sophoriside-glucoside, and peonidin caffeoyl-feruloyl-sophoroside-glucoside were not detected in ‘16-283p’ or ‘Purple-purple’ at any of the three different stages of harvesting (VS-8WAP, TIS-12WAP, and TMS-16WAP). Peonidin feruloyl-sophoroside-glucoside was present in all genotypes and at different leaf harvesting stages, including VS-8WAP, TIS-12WAP, and TMS-16WAP. Among all other genotypes and harvesting stages, ‘Purple-purple’ x TIS-12WAP exhibited the highest concentration of Peonidin feruloyl-sophoroside-glucoside. All five anthocyanin derivatives were detected in ‘2019-11-2’ at leaf harvesting related to VS-8WAP and TIS-12WAP. Moreover, cyanidin-caffeoyl-sophoroside-glucoside, cyanidin-caffeoyl-feruloyl-sophoroside-glucoside, peonidin-caffeoyl-hydroxybenzoyl-sophoriside-glucoside, and peonidin-caffeoyl-feruloyl-sophoroside-glucoside had the highest concentrations in ‘2019-11-2’ x VS-8WAP. Additionally, the levels of cyanidin-caffeoyl-sophoroside-glucoside detected in ‘2019-11-2’ at TIS-12WAP were comparable to those found at VS-8WAP. As per Zhang et al. [47], there is a positive correlation between pigment sedimentation and anthocyanin biosynthesis genes in the leaves of purple sweet potato leaves. The location of IbMYB1 genes plays a crucial role in determining the colour variation in shoots [48]. Furthermore, the expression of the IbMYB1 gene changes significantly during leaf development [47]. It is likely that the expression of this gene is affected by the growth cycle of the tubers and the number of weeks after planting. However, additional research is needed to confirm this. Our study showed that the concentrations of various anthocyanin components in the leaves of purple-fleshed sweet potato are related to growth cycle of the tubers and number of weeks since planting. Mild temperature (20 °C) and prolonged exposure to the sun enhanced the synthesis of anthocyanins in SPLs [43]. Moderate temperatures during TIS-12WAP induced the production of anthocyanins. Anthocyanins also provide photoprotection to leaf tissues which may have resulted in a high accumulation of cyanidin-caffeoyl-sophoroside-glucoside, cyanidin-caffeoyl-feruloyl-sophoroside-glucoside, peonidin-caffeoyl-hydroxybenzoyl-sophoriside-glucoside, and peonidin-caffeoyl-feruloyl-sophoroside-glucoside during VS-8WAP stage in genotype ‘2019-11-2’. Anthocyanins function as light filters during light stress [49].

#### 3.6.2. Phenolic Acids and Flavonoids

Over 20 phenolic compounds were identified and measured in the four different genotypes. The concentration of these compounds varied among the genotypes. The identified phenolic compounds include glucosyringic acid, chlorogenic acid isomers (3-O-Caffeoylquinic acid, 5-O-caffeoylquinic acid, and 4-O-caffeoylquinic acid), 1,3-dicaffeoylquinic acid (1,3-diCQA), dicaffeoylquinic acid isomers 1, 2, and 3 (diCQA 1, diCQA 2, diCQA 3), 1,3,5-tri-O-caffeoylquinic acid (1,3,5-triCQA), 3-O-caffeoyl-4-O-methylquinic acid (MCGA3), 1-O-caffeoylglucose, 2-(3,4-dihydroxyphenyl)-5-hydroxy-3,6,7-trimethoxy-4H-chromen-4-one, caffeic acid, quercetin 3-sophoroside-7-rhamnoside, quercetin 3,4′-diglucoside, quercitrin, rutin, quercetin 3-galactoside, quercetin 3-glucoside, and 6″-O-p-coumaroyltrifolin (Table 3). A detailed characterisation and MS spectral data are available in the Supplementary File (Table S6). At TIS-12WAP, the ‘Purple-purple’ genotype had the highest concentration of most phenolic compounds in its leaves. However, this genotype did not contain rutin and quercetin 3-sophoroside-7-rhamnoside. In contrast, ‘Purple-purple’ x TMS-16WAP was among the samples that had the lowest concentrations of all phenolics.

In the ‘Purple-purple’ genotype, chlorogenic acid, 5CQA, 4CQA, diCQA 1, diCQA 2, and 3-O-caffeoyl-4-O-methylquinic acid were highest at TIS-12WAP followed by ‘2019-1-1’ x TIS-12WAP. As compared to the other samples, ‘2019-11-2’ contained the highest concentrations of quercetin 3-sophoroside-7-rhamnoside at tuber initiation stage, 12 weeks after planting (12WAP). Moreover, at 12WAP stage, the highest concentration of quercetin 3.4′-diglucoside was detected in the leaves of ‘2019-11-2’ and ‘Purple-purple’ genotypes. Compared to other genotypes and their respective tuber life cycles and number of days after planting, ‘16-283P’ had the highest levels of rutin in its leaves during the VS-8WAP and during the TIS-12WAP.

The purple sweet potato leaves from Poland contain 7 phenolic compounds, including 5CQA, 3CQA, 4CQA, 3,4-diCQA, 3,5-diCQA, quercetin-3-O-galactoside, and quercetin-3-O-glucoside. Among these, 3CQA was found to be the compound with the highest concentration at 13,720 mg/kg [41]. On the other hand, the leaves of deep purple sweet potatoes from Korea had 4,5-diCQA and 3,5-diCQA as the compounds with the highest concentration (860 and 840 mg/kg, respectively) [50]. Additionally, a purple-fleshed genotype cultivar from China had the highest levels of chlorogenic acid (9850 mg/kg) and 3,5-diCQA (22,670 mg/kg) compared to 18 other cultivars from China and one USA cultivar grown in China [51]. According to our study, the purple sweet potatoes developed through Agriculture Research Council South Africa’s breeding program ‘2019-1-1’ harvested at the TIS-12WAP stage (1225.95 mg/kg), ‘2019-11-2’ harvested at the VS-8WAP stage (1337.75 mg/kg), and the local ‘Purple-purple’ at the TIS-12WAP stage (3436.47 mg/kg) showed lower 3CQA levels than those from Poland but higher than the Chinese and USA genotypes [51]. It is worth noting that the phenolic compound profiles and concentrations in Purple sweet potato leaves vary depending on the cultivars and the region of cultivation [51].

#### 3.6.3. Heat Map

**Figure 2:**
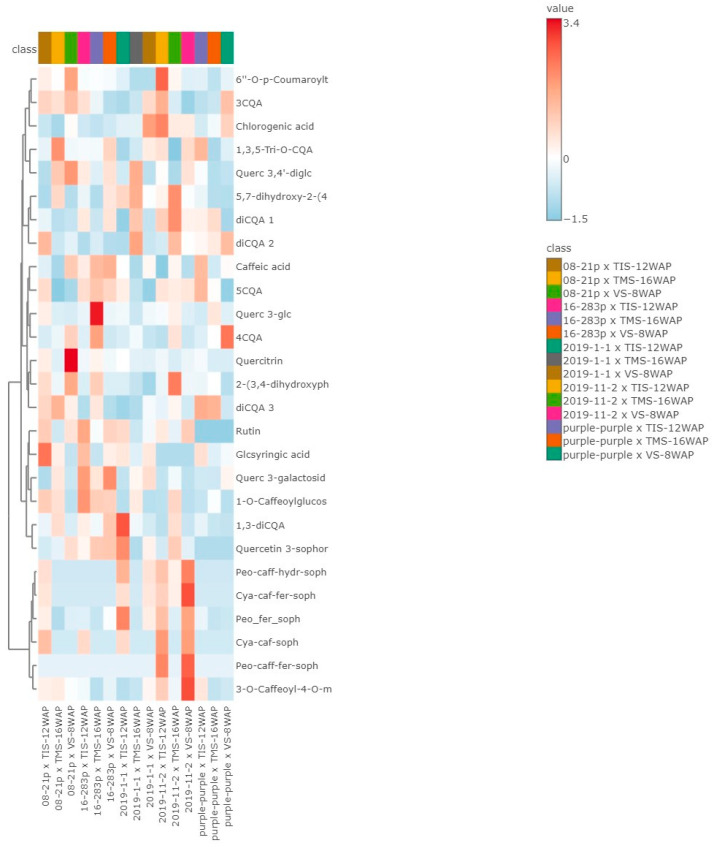
Heat map. In the map, the various phenolic compounds found in different sweet potato cultivars are coloured according to their concentration. The rows represent phenolic compounds, and the columns represent the leaves of sweet potato genotype and harvesting stage related to tuber life cycle (VS-8WAP—vegetative stage 8 weeks after planting, TIS-12WAP—tuber initiation stage 12 weeks after planting, TMS-16WAP—tuber maturation stage 16 weeks after planting). The colours red and blue indicate high and low levels, respectively.

A heat map was created based on the concentrations of phenolic compounds in all the samples. A colour block in each row represented each compound data point, with red boxes equivalent to higher levels and blue boxes indicating lower levels (Figure 2). Additionally, the heat map expressed the composition of phenolic compounds in the purple-fleshed sweet potato leaves relating to the tuber life cycle and number of days after planting of five genotypes. According to the heat map, the concentration of 1,3-diCQA and quercetin 3-sophoroside-7-rhamnoside was higher in ‘2019-1-1’ x TIS-12WAP, quercetin 3-glucoside was higher in ‘16-283P’ x TMS-16WAP. The highest concentrations of peonidin-caffeoyl-feruloyl-sophoriside-glucoside, peonidin-caffeoyl-hydroxybenzoyl-sophoriside-glucoside, cyanidin-caffeoyl-feruloyl-sophoroside-glucoside, and 3-O-caffeoyl-4-O-methylquinic acid were found in ‘2019-11-2’ x VS-8WAP.

### 3.7. Total Carotenoids (TC) and Carotenoid Components

The study found that genotype ‘16-283P’ x VS-8WAP had the highest level of TC, with a value of 7.69 mg/g dw, while genotype ‘2019-11-2’ TMS-16WAP had the lowest value at 2.75 mg/g dw. Most of the genotypes showed a similar trend of VS-8WAP > TMS-16WAP > TIS-12WAP, except for ‘Purple-purple’, as shown in Table 1. According to Hossain et al. [52], carotenoid concentration in SPLs increased from 30 to 90 days after planting and decreased from 120 to 180 days after planting. In higher plants, carotenoids and Chl b, as well as Chl a-protein complexes, are known to be involved in light-harvesting structures. Chlorophyll pigments are also protected by carotenoids from sunlight damage [53]. Furthermore, LAI was significantly reduced at TMS-16WAP, indicating that stress from shading or a large number of leaves could result in secondary metabolite synthesis. In stressed African eggplant leaves, however, carotenoids were significantly reduced, as shown by Mibei et al. [54].

The trend observed with individual carotenoids follows that of TC. The data presented are from S1 due to moderate temperature, although S2 had higher TC compared to S1. Elevated temperatures in S2 increased the carotenoids in plants to cope with climatic stress. Also, this suggest that VS-8WAP, which occurred in March with high temperature compared to April and May during TIS-12WAP and TMS-16WAP, respectively, in both seasons will favour the synthesis of carotenoids. Lutein, zeaxanthin, all-trans beta carotene, and cis beta carotene were identified and separated, as shown in Table 4. Ooko Abong et al. [55] also found the same compounds, while Drapal et al. [56] additionally identified violaxanthin. The levels of individual carotenoids were influenced by the genotype x harvesting stage and main effects (*p* < 0.001). The highest lutein content (128.35 mg/100 g dw), all-trans β-carotene (60.20 mg/100 g dw), zeaxanthin (46.83 mg/100 g dw), and cis-β-carotene (9.20 mg/100g dw) were found in genotype ‘08-21P’ x VS-8WAP, while the least were obtained in genotype ‘2019-1-1’ x TMS-16WAP (52.43 mg/100 g dw), (9.11 mg/100 g dw), (9.11 mg/100 g dw) and (3.07 mg/100 g dw) of lutein, all trans-β-carotene, zeaxanthin and cis-β-carotene, respectively, as presented in Table 4.

Lutein was the predominant compound, making up 56.01% of the TC, followed by all-trans-β-carotene, which contributed 26.99%. According to Ooko Abong et al. [55] and Bolanos [27], lutein is also an abundant compound. However, Phahlane et al. [25] reported that all trans-β-carotene was the major compound accumulated in the leaves of orange flesh sweet potatoes. The study observed that the sweet potato plant growth stage VS-8WAP had the highest accumulation of cis-β-carotene, followed by TIS-12WAP and TMS-16WAP. With zeaxanthin, the order was VS-8WAP, TMS-16WAP, and TIS-12WAP, except for the genotype ‘08-21P’, where it was VS-8WAP, TIS-12WAP, and TMS-16WAP. With trans-β-carotene, the order was VS-8WAP, TIS-12WAP, and TMS-16WAP, except for the genotype ‘16-283P’, where it was VS-8WAP, TMS-16WAP, and TIS-12WAP. Lutein followed the order VS-8WAP, TIS-12WAP, and TMS-16WAP in the genotypes ‘Purple-purple’, ‘08-21P’, and ‘2019-1-1’, whereas in ‘2019-11-2’ and ‘16-283P’, it was TMS-16WAP, VS-8WAP, and TIS-12WAP.

The accumulation of carotenoid-derived metabolites can depend on the environment, affecting plant growth, development, and signalling processes [57]. Therefore, a high accumulation was observed during VS-8WAP. In grapevine, β-carotene increased with an increase in temperature, while lutein showed the opposite trend. Similarly, kale and spinach showed high concentrations of lutein and β-carotene at temperatures under 30 °C and 10 °C, respectively. In this study, β-carotene was high during the vegetative stage and lower in the last harvest with lower temperatures [58].

### 3.8. Antioxidant Activities

There was a significant effect observed when genotype, harvesting stage, and season were compared (*p* < 0.001, Table S6 of Supplementary File). The total phenolic content (TPC) and ferric-reducing antioxidant power (FRAP) showed a similar trend. Among all genotypes, the best antioxidant power (FRAP) value (53.92 TEAC mM/g dw) was obtained in TIS-12WAP of ‘2019-11-2’, while the lowest value (15.17 TEAC mM/g dw) was observed in TMS-16WAP of ‘2019-1-1’ (Table 5). The trend observed was TIS-12WAP > VS-8WAP > TMS-16WAP in all genotypes. However, in a study conducted by Suárez et al. [40], high FRAP was obtained in fresh leaves of cultivar Shangshu no. 19 harvested at HP3 in China, which could be attributed to the environmental conditions and the stage at which the leaves were harvested.

Table 5 shows the ABTS scavenging activity of SPLs of five purple-fleshed genotypes. Genotype, harvesting stage, and season significantly influenced the antioxidant activity with ABTS assay in SPLs (*p* < 0.001) (Table S6 of Supplementary File). The genotype ‘2019-11-2’ at VS-8WAP stage exhibited the highest ABTS scavenging activity (0.20 IC_50_ mg/mL), which was not significantly different from that of ‘2019-11-2’ x TIS-12WAP. According to Jia et al. [18], the strength of AA increases as the IC_50_ value decreases. The genotype ‘2019-1-1’ x TMS-16WAP exhibited a weaker ABTS with an IC_50_ value of 1.31 mg/mL. Phahlane et al. [25] reported ABTS values ranging between 3.43 and 4.60 mg/mL in the leaves of orange flesh sweet potato local cultivars weaker compared to the present study. It is possible that the variation can be attributed to the harvesting stage, genotype, and environmental conditions. The IC_50_ of ABTS was higher in SPLs harvested during TIS-12WAP and lower during the TMS-16WAP. Additionally, it was significantly higher in the genotype ‘2019-11-2’ with purple-coloured leaves.

An important observation was made regarding the effect of genotype, harvesting stage, and season (*p* < 0.001), as well as the main effects, as shown in Table S6 of Supplementary File. According to Table 5, ‘2019-11-2’ demonstrated a strong ability to scavenge DPPH radicals in SPLs harvested during VS-8WAP (0.66 IC_50_ mg/mL) and TIS-12-WAP (0.58 IC_50_ mg/mL) during both seasons, with no significant difference between the two. This value is comparable to the IC_50_ reported by Zhang et al. [11], which was 0.758 mg/mL. In the current study, the combination of ‘08-21P’ at TMS-16WAP harvesting stage showed the weakest DPPH with an IC_50_ of 2.54 mg/mL. On the other hand, the DPPH values of the leaves of South African orange fleshed local cultivars ranged from 3.51 to 5.21 mg/mL, as reported by Phahlane et al. [25]. The reason for this outcome may be the type of plant or the timing of when the leaves were harvested. The results showed that the leaves of ‘Purple-purple’, ‘08-21P’, ‘2019-1-1’, and ‘2019-11-2’ genotypes at 16WAP demonstrated lower DPPH scavenging activity. Leaves with high anthocyanin content have been found to possess strong DPPH scavenging activity [59], hence a potent in genotype ‘2019-11-2’. Abiotic stress triggers the production of reactive oxygen species (ROS), which prompts plants to regulate their metabolic enzymes to produce antioxidants. When the production of free radicals exceeds that of free radical scavengers (antioxidants), the plant undergoes oxidative stress [60]. The sweet potato leaf extracts of these genotypes indicate the presence of antioxidant properties with the ability to protect the plant against oxidative stress and the ability to provide physiological defence against oxidative and free-radical-mediated reactions promoting health benefits to consumers [2]. Furthermore, SPLs could serve as a natural source of natural antioxidants in this growing interest over synthetic antioxidants because it will be safer and there are no health risk issues. In a recent study by Krochmal-Marczak et al. [41], the total antioxidant activity varied across different leaf samples harvested at three stages of growth from various cultivars as compared to our present study. The correlation between individual compounds and the antioxidant power (FRAP) are shown in Figure S3A of Supplementary File. Cyanidin-caffeoyl-sophoroside-glucoside (r = 0.60, *p* < 0.05) displayed the strongest correlation with the FRAP value, followed by cyanidin-caffeoyl-feruloyl-sophoroside-glucoside (r = 0.58, *p* < 0.05), peonidin-caffeoyl-hydroxybenzoyl-sophoriside-glucoside (r = 0.56, *p* < 0.05), peonidin feruloyl-sophoroside-glucoside (r = 0.55, *p* < 0.05), peonidin-caffeoyl-feruloyl-sophoroside-glucoside (r = 0.54, *p* < 0.05), and chlorogenic acid (r = 0.42, *p* < 0.05). The highest correlation with the ABTS scavenging activity (Figure S3B of Supplementary File) were exhibited by peonidin-caffeoyl-feruloyl-sophoroside-glucoside (r = 0.59, *p* < 0.05), followed by cyanidin-caffeoyl-sophoroside-glucoside (r = 0.52, *p* < 0.05), 5CQA (r = 0.50, *p* < 0.05), and rutin (r = 0.40, *p* < 0.05), whereas for DPPH scavenging activity, the highest correlation (Figure S3C of Supplementary File) was with cyanidin-caffeoyl-sophoroside-glucoside (r = 0.67, *p* < 0.05), followed by peonidin-caffeoyl-hydroxybenzoyl-sophoriside-glucoside (r = 0.56, *p* < 0.05), peonidin feruloyl-sophoroside-glucoside (r = 0.54, *p* < 0.05), cyanidin-caffeoyl-feruloyl-sophoroside-glucoside (r = 0.47, *p* < 0.05), and rutin (r = 0.40, *p* < 0.05). The antioxidant effects of phenolic compounds had been associated with the number of hydroxy groups present in their structures [61]. Moriyama et al. [62] reported that the superoxide anion-scavenging activity of acylated anthocyanins varied depending on the activity of each corresponding deacylated compound. Phenolic hydroxyl group in anthocyanins contribute substantially to scavenging reactive oxygen radicals [63].

### 3.9. Mineral Contents

The accumulation of minerals in purple-fleshed sweet potato genotypes was influenced by the interaction of the genotype x harvesting stage x season and all the main effects at *p* < 0.001 (Table S7 of Supplementary File). Mineral contents were accumulated as follows: genotype ‘08-21P’ x VS-8WAP contained high content of N (4.62% dw), K (3.52% dw), and Cu (2.14 mg/100 g dw); ’08-21P’ x TMS-16WAP high Fe (35.07 mg/100 g dw); ‘2019-11-2’ x VS-8WAP had high P (0.59% dw), ‘2019-11-2’ x TMS-16WAP high Mg (0.46% dw); Ca (1.15 mg/100 g dw) and Mn (16.41 mg/100 g dw) were high in ‘2019-1-1’ x TMS-16WAP; ‘Purple-purple’ x TMS-16WAP contain high B (4.73 mg/100 g dw). Zn was high in leaves of ‘08-21P’ x VS-8WAP (4.23 mg/100 g dw) and ‘2019-11-2’ x VS-8WAP (4.19 mg/100 g dw). K and Fe were the predominant elements accumulated in SPLs within the macro and micro mineral group, respectively. K is essential for the human body to function properly and for the plants to withstand water stress [64]. On the other hand, Fe regulates plant processes [65].

SPLs in five purple-fleshed genotypes accumulated mineral contents differently during different harvesting stages. K was high in SPLs harvested during VS-8WAP, whereas contents of Fe were higher in SPLs harvested at TMS-16WAP except for genotype ‘2019-11-2’ and ‘2019-1-1’ in both seasons in all genotypes. Suárez et al. [40] determined mineral content during three harvesting periods. These mineral contents were higher at the first harvest in agreement with the findings in this present study. High accumulation of Fe at H1 contradict with the high content in ’Purple-purple’, ‘08-21P’ and ‘16-283P’ during TMS-16WAP. K and Fe plays a role in plant growth and development, hence high accumulation during the vegetative stage [66,67]. The accumulation of minerals can be induced by several factors. For instance, under biotic stress, plants become activated and generate cellular responses once they detect stress [68]. Fe alleviates abiotic stresses through its ability to exist in two oxidation states as Fe^+3^ and Fe^+2^ whereas K offers reactive oxygen species (ROS) defence and hinders the accumulation of ROS [66,69,70]. Therefore, under harsh environmental conditions sweet potatoes need to be supplemented with minerals to avoid plant stress and to maintain the synthesis of secondary metabolites. The mineral analysis obtained in our study is comparable to the results obtained by Suárez et al. [40] and Sun et al. [42]. SPLs contain nutritional compounds found in leafy vegetables, such as spinach, kale, Chinese cabbage, black nightshade, pigweed, jaw’s mallow, cowpea, pumpkin, and spider flower. Therefore, sweet potatoes are a good competitive leafy vegetable crop that is good for human consumption.

## 4. Conclusions

This study investigated the effects of tuber life cycles and days after planting on leaf anthocyanin and phenolic acids, predominantly chlorogenic acids derivatives and micronutrients. This helps in harvesting the correct leaf stage for consumption or for use as a functional ingredient. The use of sweet potato leaves as leafy vegetables is growing globally and is already very common in Asian countries; but, due to their functional properties, it is likely to become a niche market in the future. It would also be ideal to recommend purple-fleshed sweet potato genotypes that can be used as leafy vegetables in Southern Africa. The high anthocyanin and caffeoylquinic acid derivative content of purple-fleshed sweet potato genotypes ‘2019-11-2’ and ‘Purple-purple’ warrants their commercialisation as leafy vegetables. In the future, it will be necessary to investigate the palatability and antinutritive components of the leaves in relation to the tuber life cycle and days after planting.

## Figures and Tables

**Table 1:** The level of TPC and TC in leaves of five purple-fleshed genotypes harvested at different harvesting stages.

Genotype X Harvesting Stage	TPC CAE mg/g dw			Total Carotenoids mg/g dw		
	S1	S2	Means	S1	S2	Means
Purple-purple X VS-8WAP	169.08 ± 1.81 ^j^	125.5 ± 1.33 ^h^	147.3 ± 23.89 ^j^	5.08 ± 0.12 ^c^	7.85 ± 0.05 ^c^	6.465 ± 1.52 ^d^
Purple-purple X TIS-12WAP	224.76 ± 2.35 ^g^	150.4 ± 2.61 ^f^	190.1 ± 38.06 ^f^	4.53 ± 0.08 ^d^	4.03 ± 0.03 ^ij^	4.278 ± 0.28 ^g^
Purple-purple X TMS-16WAP	133.39 ± 1.89 ^m^	115.7 ± 3.52 ^i^	124.5 ± 10.02 ^m^	4.14 ± 0.03 ^e^	4.43 ± 0.18 ^h^	4.283 ± 0.19 ^g^
08-21P X VS-8WAP	256.14 ± 0.90 ^e^	104.4 ± 1.33 ^k^	180.3 ± 83.13 ^h^	6.18 ± 0.62 ^b^	8.02 ± 0.0 ^b^	7.100 ± 1.08 ^b^
08-21P X TIS-12WAP	285.75 ± 1.80 ^c^	183.8 ± 1.33 ^c^	234.8 ± 55.86 ^c^	6.35 ± 0.19 ^b^	7.03 ± 0.04 ^e^	6.863 ± 0.58 ^c^
08-21P X TMS-16WAP	125.16 ± 3.24 ^n^	94.5 ± 1.74 ^l^	109.8 ± 19.64 ^n^	3.22 ± 0.13 ^f^	7.37 ± 0.124 ^d^	5.125 ± 2.09 ^e^
2019-11-2 X VS-8WAP	301.24 ± 0.59 ^a^	177.7 ± 1.33 ^d^	239.4 ± 67.63 ^b^	3.48 ± 0.03 ^f^	5.92 ± 0.04 ^f^	4.700 ± 1.34 ^f^
2019-11-2 X TIS-12WAP	282.41 ± 2.56 ^d^	242.1 ± 2.5 ^a^	262.2 ± 22.22 ^a^	4.04 ± 0.13 ^e^	3.38 ± 0.05 ^l^	3.970 ± 0.12 ^h^
2019-11-2 X TMS-16WAP	202.23 ± 2.01 ^h^	170.5 ± 1.81 ^e^	186.3 ± 17.49 ^g^	2.12 ± 0.10 ^h^	3.90 ± 0.06 ^jk^	2.752 ± 0.70 ^k^
16-283P X VS-8WAP	193.16 ± 0.63 ^i^	131.3 ± 0.50 ^g^	162.2 ± 33.87 ^i^	6.75 ± 0.07 ^a^	8.64 ± 0.18 ^a^	7.693 ± 1.04 ^a^
16-283P X TIS-12WAP	252.70 ± 2.82 ^f^	192.8 ± 0.87 ^b^	222.7 ± 32.87 ^e^	4.37 ± 0.38 ^de^	3.85 ± 0.12 ^k^	4.190 ± 0.31 ^gh^
16-283P X TMS-16WAP	164.06 ± 0.99 ^k^	108.7 ± 3.92 ^j^	136.4 ± 30.41 ^k^	2.63 ± 0.18 ^g^	4.01 ± 0.06 ^ijk^	3.237 ± 0.68 ^j^
2019-1-1 X VS-8WAP	138.72 ± 0.87 ^l^	125.8 ± 1.51 ^h^	132.3 ± 7.15 ^l^	3.11 ± 0.03 ^f^	7.25 ± 0.07 ^d^	5.182 ± 2.27 ^e^
2019-1-1 X TIS-12WAP	295.10 ± 0.33 ^b^	168.1 ± 1.32 ^e^	231.9 ± 69.79 ^d^	2.75 ± 0.17 ^g^	4.09 ± 0.10 ^i^	3.892 ± 1.27 ^ij^
2019-1-1 X TMS-16WAP	107.87 ± 0.87 ^o^	62.1 ± 1.33 ^m^	85.0 ± 25.11 ^o^	2.11 ± 0.03 ^h^	5.04 ± 0.12 ^g^	3.100 ± 1.09 ^i^
LSD	2.87	3.29		0.38	0.17	

Data are means and standard deviation (*n* = 3). Small letters within the same column represent the significant differences between genotypes and harvesting stages. S1—season 1, S2—season 2, VS-8WAP—vegetative stage 8 weeks after planting, TIS-12WAP—tuber initiation stage 12 weeks after planting, TMS-16WAP—tuber maturation stage 16 weeks after planting.

**Table 2:** Comparison of anthocyanin compounds detected in the leaves of different purple-fleshed sweet potato genotypes at different stages of harvesting.

Molecular Formula	Compound	Concentrations in mg/kg in the Solid versus Neochlorogenic Acid (3CQA) Calibration Curve														
		16-283p x VS-8WAP	16-283p x TIS-12WAP	16-283p x TMS-16WAP	08-21p x VS-8WAP	08-21p x TIS-12WAP	08-21p x TMS-16WAP	2019-1-1 x VS-8WAP	2019-1-1 x TIS-12WAP	2019-1-1 x TMS-18WAP	2019-11-2 x VS-8WAP	2019-11-2 x TIS-12WAP	2019-11-2 x TMS-16WAP	Purple-purple x VS-8WAP	Purple-purple x TIS-12WAP	Purple-purple x TMS-16WAP
C_42_H_45_O_24_	Cyanidin-caffeoyl-sophoroside-glucoside	nd	9.86 ± 1.72 ^c^	nd	nd	13.46 ± 0.41 ^b^	nd	nd	14.56 ± 0.43 ^b^	nd	17.64 ± 0.87 ^a^	18.13 ± 0.74 ^a^	nd	nd	nd	nd
C_44_H_49_O_24_	Peonidin feruloyl-sophoroside-glucoside	66.30 ± 0.47 ^h^	66.48 ± 0.66 ^g^	28.28 ± 1.34 ^m^	64.72 ± 1.35 ^i^	87.35 ± 1.99 ^f^	14.52 ± 0.44 ^o^	88.76 ± 1.25 ^e^	267.10 ± 3.13 ^b^	44.15 ± 3.51 ^j^	161.74 ± 4.63 ^c^	134.67 ± 1.80 ^d^	43.74 ± 2.09 ^k^	32.01 ± 3.19 ^l^	298.52 ± 444.01 ^a^	27.29 ± 0.76 ^n^
C_52_H_53_O_27_	Cyanidin-caffeoyl-feruloyl-sophoroside-glucoside	nd	nd	nd	nd	13.68 ± 0.30 ^d^	nd	13.32 ± 0.53 ^d^	21.56 ± 1.28 ^c^	nd	41.51 ± 3.33 ^a^	27.51 ± 0.61 ^b^	11.17 ± 0.89 ^d^	nd	nd	nd
C_50_H_51_O_26_	Peonidin-caffeoyl-hydroxybenzoyl-sophoriside-glucoside	nd	nd	nd	nd	13.99 ± 1.08 ^d^	nd	13.43 ± 0.54 ^d^	22.83 ± 2.15 ^c^	nd	45.24 ± 1.61 ^a^	29.13 ± 0.40 ^b^	10.43 ± 0.29 ^e^	nd	nd	nd
C_53_H_55_O_27_	Peonidin caffeoyl-feruloyl-sophoroside-glucoside	nd	nd	nd	nd	nd	nd	nd	nd	nd	24.47 ± 1.67 ^a^	21.16 ± 0.14 ^b^	nd	nd	nd	nd

Data are means and standard deviation (*n* = 3). Means followed by the same letter within the row are not significantly different (*p* < 0.05), each of the samples was replicated three times, and the results are expressed as mean ± standard deviation. nd (No anthocyanin detected). VS-8WAP—vegetative stage 8 weeks after planting, TIS-12WAP—tuber initiation stage 12 weeks after planting, TMS-16WAP—tuber maturation stage 16 weeks after planting.

**Table 3:** UPLC-QTOF/MS analysis of major phenolic compounds (chlorogenic derivatives and flavonoids) detected in sweet potatoes leaves.

RT/ [M-H]-	Molecular Formula	Compound	Concentrations in mg/Kg in the Solid versus Neochlorogenic Acid (3CQA) Calibration Curve														
			16-283p x VS-8WAP	16-283p x TIS-12WAP	16-283p x TMS-16WAP	08-21p x VS-8WAP	08-21p x TIS-12WAP	08-21p x TMS-16WAP	2019-1-1 x VS-8WAP	2019-1-1 x TIS-12WAP	2019-1-1 x TMS-18WAP	2019-11-2 x VS-8WAP	2019-11-2 x TIS-12WAP	2019-11-2 x TMS-16WAP	Purple-purple x VS-8WAP	Purple-purple x TIS-12WAP	Purple-purple x TMS-16WAP
3.77/ 359.09	C_15_H_20_O_10_	Glucosyringic acid; Syringin 4-O-beta-glucoside	48.62 ± 1.23 ^d^	49.82 ± 0.82 ^c^	24.03 ± 0.58 ^i^	15.57 ± 1.51 ^m^	68.81 ± 1.83 ^b^	34.21 ± 2.49 ^h^	36.39 ± 0.62 ^g^	36.90 ± 0.74 ^f^	21.92 ± 0.25 ^j^	11.66 ± 0.27 ^n^	11.65 ± 0.83 ^o^	16.52 ± 0.59 ^l^	38.86 ± 1.55 ^e^	113.57 ± 189.43 ^a^	21.91 ± 1.21 ^k^
4.44/ 353.08	C_16_H_18_O_9_	Chlorogenic acid (3CQA)	1023.68 ± 23.95 ^d^	994.74 ± 28.43 ^e^	300.43 ± 7.57 ^k^	722.20 ± 43.38 ^g^	612.18 ± 10.83 ^h^	54.72 ± 2.16 ^o^	580.94 ± 3.08 ^i^	1225.95 ± 11.91 ^c^	121.48 ± 5.05 ^n^	1337.75 ± 26.14 ^b^	816.14 ± 13.65 ^f^	161.24 ± 5.29 ^l^	502.71 ± 7.06 ^j^	3436.47 ± 5705.74 ^a^	122.17 ± 1.46 ^m^
5.08/ 341.09	C_15_H_18_O_9_	1-O-Caffeoylglucose	84.71 ± 2.17 ^j^	86.20 ± 2.17 ^i^	60.32 ± 1.58 ^l^	116.61 ± 6.07 ^d^	86.60 ± 0.95 ^h^	50.93 ± 1.62 ^n^	102.47 ± 1.09 ^g^	137.02 ± 4.05 ^b^	64.59 ± 1.63 ^k^	106.21 ± 2.48 ^e^	119.32 ± 2.35 ^c^	55.91 ± 1.61 ^m^	102.61 ± 0.72 ^f^	396.52 ± 657.99 ^a^	47.82 ± 0.99 ^o^
5.11/ 359.08	C_18_H_16_O_8_	2-(3,4-dihydroxyphenyl)-5-hydroxy-3,6,7-trimethoxy-4H-chromen-4-one	207.34 ± 8.11 ^c^	205.17 ± 27.17 ^d^	45.21 ± 1.41 ^j^	79.95 ± 5.30 ^h^	406.69 ± 8.72 ^b^	25.17 ± 1.98 ^l^	123.66 ± 4.47 ^g^	203.51 ± 5.99 ^e^	15.28 ±1.33 ^n^	24.95 ± 1.27 ^m^	70.97 ± 3.81 ^i^	10.20 ± 1.00 ^o^	128.03 ± 5.66 ^f^	1091.55 ± 1826.59 ^a^	29.25 ± 2.19 ^k^
5.29/ 707.18	C_16_H_18_O_9_	Chlorogenic acid	1366.14 ± 98.61 ^c^	1290.55 ± 206.01 ^d^	13.22 ± 0.68 ^k^	85.91 ± 3.44 ^i^	1109.61 ± 75.64 ^e^	0.27 ± 0.47 ^o^	78.60 ± 2.86 ^j^	2064.07 ± 103.18 ^b^	2.08 ± 2.22 ^n^	578.08 ± 46.32 ^g^	881.00 ± 59.74 ^f^	11.60 ± 1.84 ^l^	88.03 ± 27.37 ^h^	6688.82 ± 11,201.25 ^a^	3.13 ± 1.29 ^m^
5.29/ 353.09	C_16_H_18_O_9_	Chlorogenic acid (5CQA)	3496.49 ± 153.12 ^c^	3314.37 ± 244.52 ^e^	545.67 ± 15.12 ^k^	1284.58 ± 67.53 ^h^	3341.79 ± 64.01 ^d^	128.76 ± 9.86 ^o^	1035.82 ± 16.67 ^j^	4243.55 ± 75.56 ^b^	234.45 ± 10.73 ^n^	3137.42 ± 86.85 ^g^	3141.00 ± 89.10 ^f^	419.77 ± 18.94 ^l^	1041.20 ± 25.21 ^i^	10,916.61 ± 18,124.68 ^a^	263.62 ± 17.55 ^m^
5.62/ 179.03	C_9_H_8_O_4_	Caffeic acid	663.70 ± 8.14 ^e^	655.66 ± 56.66 ^f^	629.45 ± 21.20 ^g^	802.95 ± 61.90 ^d^	436.15 ± 9.07 ^j^	163.39 ± 2.43 ^o^	619.37 ± 3.48 ^h^	555.08 ± 23.90 ^i^	179.06 ± 3.35 ^n^	2023.06 ± 106.85 ^a^	1067.31 ± 16.11 ^c^	416.25 ± 19.33 ^k^	398.78 ± 25.62 ^l^	1668.72 ± 2769.16 ^b^	265.09 ± 6.40 ^m^
5.73/ 353.09	C_16_H_18_O_9_	Chlorogenic acid (4CQA)	213.92 ± 3.80 ^d^	203.40 ± 5.13 ^e^	76.25 ± 2.81 ^k^	76.95 ± 4.15 ^j^	253.11 ± 4.71 ^c^	25.43 ± 0.86 ^o^	119.73 ± 0.81 ^h^	266.47 ± 7.14 ^b^	27.32 ± 1.06 ^n^	195.83 ± 4.58 ^f^	123.96 ± 0.64 ^g^	45.82 ± 1.30 ^l^	91.28 ± 1.87 ^i^	557.67 ± 927.91 ^a^	45.28 ± 0.93 ^m^
6.02/ 771.20	C_33_H_40_O_21_	Quercetin 3-sophoroside-7-rhamnoside	4.65 ± 0.45 ^de^	3.80 ± 0.33 ^e^	4.48 ± 0.17 ^de^	9.81 ± 0.47 ^cd^	14.38 ± 1.81 ^c^	1.76 ± 0.88 ^e^	4.55 ± 0.93 ^de^	9.85 ± 0.26 ^cd^	0	139.93 ± 8.30 ^a^	132.78 ± 3.37 ^a^	44.35 ± 3.57 ^b^	0	0	0
6.28/ 625.14	C_27_H_30_O_17_	Quercetin 3,4′-diglucoside	43.93 ± 2.49 ^c^	41.24 ± 1.23 ^c^	40.21 ± 2.60 ^c^	960.00 ± 46.96 ^abc^	1843.07 ± 36.94 ^abc^	516.53 ± 14.18 ^bc^	196.69 ± 4.92 ^bc^	349.98 ± 6.68 ^bc^	84.90 ± 2.55 ^c^	4387.54 ± 86.93 ^a^	3558.71 ± 22.77 ^ab^	1679.59 ± 50.85 ^abc^	211.30 ± 2.42 ^bc^	2672.41 ± 4434.14 ^abc^	266.33 ± 5.68 ^bc^
6.48/ 367.10	C_17_H_20_O_9_	3-O-Caffeoyl-4-O-methylquinic acid	21.28 ± 17.67 ^k^	27.56 ± 0.42 ^h^	12.16 ± 0.79 ^n^	29.80 ± 2.18 ^g^	36.64 ± 1.55 ^e^	5.60 ± 0.58 ^o^	34.42 ± 1.72 ^f^	116.20 ± 5.65 ^b^	22.45 ± 0.26 ^j^	88.22 ± 2.26 ^c^	70.84 ± 1.25 ^d^	26.26 ± 0.88 ^i^	17.62 ± 0.25 ^l^	144.79 ± 240.55 ^a^	14.46 ± 0.17 ^m^
6.94/ 609.15	C_27_H_30_O_16_	Rutin	523.04 ± 5.86 ^a^	499.00 ± 22.46 ^ab^	336.23 ± 33.62 ^c^	31.91 ± 1.21 ^e^	38.74 ± 16.65 ^e^	16.66 ± 0.51 ^e^	201.12 ± 68.26 ^d^	346.79 ± 110.04 ^c^	110.66 ± 1.11 ^de^	387.42 ± 6.33 ^bc^	442.93 ± 55.85 ^abc^	168.54 ± 3.77 ^d^	0	0	0
7.12/ 463.09	C_21_H_20_O_12_	Quercetin 3-galactoside	829.10 ± 23.77 ^i^	778.36 ± 26.89 ^j^	455.13 ± 10.78 ^m^	362.90 ± 23.20 ^o^	1066.87 ± 12.09 ^g^	442.59 ± 7.74 ^n^	695.64 ± 11.68 ^k^	1303.50 ± 31.28 ^d^	532.94 ± 3.88 ^l^	1114.58 ± 24.86 ^f^	1280.30 ± 37.36 ^e^	856.03 ± 281.72 ^h^	1457.26 ± 596.22 ^c^	4440.52 ± 7988.66 ^a^	1631.68 ± 37.63 ^b^
7.19/ 463.09	C_21_H_20_O_12_	Quercetin 3-glucoside	1841.13 ± 60.71 ^g^	1755.14 ± 84.36 ^h^	977.69 ± 13.20 ^o^	1205.94 ± 58.73 ^l^	3148.21 ± 31.66 ^b^	1338.57 ± 8.01 ^j^	1989.18 ± 4.83 ^f^	2828.49 ± 58.88 ^c^	1192.43 ± 14.03 ^m^	2044.06 ± 36.91 ^e^	2182.17 ± 44.90 ^d^	1016.90 ± 285.66 ^n^	1451.40 ± 585.40 ^i^	9064.57 ± 15,266.96 ^a^	1318.79 ± 507.70 ^k^
7.41/ 515.12	C_25_H_24_O_12_	1,3-Dicaffeoylquinic acid	5628.98 ± 148.03 ^b^	5406.35 ± 217.99 ^c^	2581.02 ± 35.47 ^e^	1784.22 ± 92.81 ^g^	3371.92 ± 74.59 ^d^	465.04 ± 4.23 ^l^	156.95 ± 2.08 ^o^	971.08 ± 21.29 ^k^	252.23 ± 6.65 ^n^	1194.78 ± 30.87 ^j^	2153.33 ± 84.09 ^f^	459.68 ± 10.96 ^m^	1562.58 ± 26.21 ^h^	16,752.67 ± 28,022.12 ^a^	1213.85 ± 21.59 ^i^
7.57/ 447.09	C_21_H_20_O_11_	Quercitrin	186.44 ± 3.56 ^d^	180.64 ± 8.33 ^f^	94.52 ±14.08 ^n^	89.35 ± 5.12 ^o^	294.47 ± 8.73 ^b^	138.55 ± 1.51 ^j^	138.98 ± 3.10 ^i^	222.93 ±0.64 ^c^	146.41 ± 4.84 ^h^	167.32 ± 6.83 ^g^	182.41 ± 2.60 ^e^	123.02 ± 2.87 ^k^	121.62 ± 1.56 ^m^	591.06 ± 986.92 ^a^	122.19 ± 0.87 ^l^
7.60/ 515.12	C_25_H_24_O_12_	Dicaffeoylquinic acid isomer 1 (diCQA 1)	5807.08 ± 195.76 ^d^	5530.04 ± 270.98 ^e^	1051.02 ± 333.66 ^k^	2555.37 ± 137.16 ^h^	3724.60 ± 26.86 ^g^	217.49 ± 10.74 ^o^	2487.34 ± 28.98 ^i^	11217.07 ± 215.35 ^b^	720.16 ± 15.32 ^m^	5141.49 ± 80.57 ^f^	6709.48 ± 98.04 ^c^	947.11 ± 7.73 ^l^	1579.57 ± 9.63 ^j^	16,302.09 ± 27,303.72 ^a^	615.24 ± 15.97 ^n^
7.86/ 477.10	C_22_H_22_O_12_	5,7-dihydroxy-2-(4-hydroxy-3-methoxyphenyl)-3-{[3,4,5-trihydroxy-6-(hydroxymethyl)oxan-2-yl]oxy}-4H-chromen-4-one	51.04 ± 2.42 ^ab^	48.36 ± 1.47 ^ab^	22.90 ± 0.20 ^ab^	17.03 ± 0.48 ^b^	151.68 ± 3.89 ^a^	81.95 ± 1.53 ^ab^	39.52 ± 1.33 ^ab^	83.09 ± 1.15 ^ab^	74.73 ± 7.34 ^ab^	39.02 ± 1.10 ^ab^	64.57 ± 0.75 ^ab^	86.18 ± 0.33 ^ab^	17.26 ± 0.90 ^b^	100.64 ± 168.14 ^ab^	17.56 ± 0.56 ^b^
8.13/ 515.12	C_25_H_24_O_12_	Dicaffeoylquinic acid isomer 2 (diCQA 2)	1183.62 ± 50.88 ^bc^	1111.71 ± 40.60 ^bc^	191.32 ± 3.51 ^c^	311.99 ± 17.26 ^bc^	506.51 ± 12.19 ^bc^	22.63 ± 2.36 ^c^	158.85 ± 4.47 ^c^	1099.61 ± 37.26 ^bc^	57.30 ± 5.06 ^c^	2848.34 ± 1150.57 ^a^	1842.83 ± 64.43 ^ab^	497.90 ± 15.91 ^bc^	83.23 ± 25.71 ^c^	977.54 ± 1637.79 ^bc^	35.21 ± 10.06 ^c^
8.67/ 515.12	C_25_H_24_O_12_	Dicaffeoylquinic acid isomer 3 (diCQA 3)	115.49 ± 1.30 ^ab^	116.96 ± 9.02 ^ab^	37.17 ± 1.28 ^ab^	47.88 ± 3.52 ^ab^	63.68 ± 1.79 ^ab^	5.38 ± 0.94 ^b^	26.93 ± 0.36 ^b^	108.30 ± 3.05 ^ab^	10.94 ± 1.24 ^b^	261.64 ± 10.50 ^a^	234.66 ± 2.62 ^ab^	46.01 ± 0.96 ^ab^	17.27 ± 0.65 ^b^	176.80 ± 296.17 ^ab^	8.85 ± 1.30 ^b^
9.40/ 677.15	C_34_H_30_O_15_	1,3,5-Tri-O-caffeoylquinic acid	49.05 ± 0.13 ^d^	45.68 ± 2.67 ^e^	45.53 ± 1.02 ^f^	45.39 ± 1.66 ^g^	41.69 ± 1.63 ^i^	6.88 ± 0.50 ^o^	41.26 ± 1.57 ^j^	143.50 ± 5.75 ^b^	23.54 ^l^± 0.80 ^l^	69.31 ± 1.13 ^c^	44.65 ± 1.42 ^h^	12.11 ± 0.68 ^n^	27.10 ± 1.25 ^k^	239.29 ± 405.51 ^a^	15.31 ± 0.15 ^m^
9.53/ 593.13	C_30_H_26_O_13_	6″-O-p-Coumaroyltrifolin	33.04 ± 0.61 ^h^	35.38 ± 3.34 ^f^	35.66 ± 2.32 ^e^	101.15 ± 3.83 ^d^	33.89 ± 1.19 ^g^	26.73 ± 1.24 ^i^	0.31 ± 0.53 ^n^	1.49 ± 1.09 ^m^	nd	14.90 ± 0.93 ^j^	9.12 ± 0.65 ^k^	3.03 ± 0.81 ^l^	249.14 ± 12.78 ^b^	717.03 ± 1201.93 ^a^	104.39 ± 8.11 ^c^

Data are means and standard deviation (*n* = 3). Small letters within the same row represent the significant differences between genotypes and harvesting stages. VS-8WAP—vegetative stage 8 weeks after planting, TIS-12WAP—tuber initiation stage 12 weeks after planting, TMS-16WAP—tuber maturation stage 16 weeks after planting. Means followed by the same letter within the row are not significantly different (*p* < 0.05), each of the samples was replicated three times, and the results are expressed as mean ± standard deviation.

**Table 4:** The level of individual carotenoids isolated in sweet potato leaves harvested during three different stages.

Genotypes x Harvesting Stages	Lutein mg/100 g dw	All Trans-β-carotene mg/100 g dw	Zeaxanthin mg/100 g dw	Cis-β-carotene mg/100 g dw
Purple-purple X VS-8WAP	100.22 ± 0.38 ^b^	56.94 ± 0.26 ^b^	33.60 ± 0.16 ^c^	7.11 ± 0.00 ^b^
Purple-purple X TIS-12WAP	76.15 ± 0.09 ^f^	22.83 ± 0.07 ^i^	9.85 ± 0.01 ^j^	5.68 ± 0.00 ^f^
Purple-purple X TMS-16WAP	63.59 ± 0.00 ^k^	14.02 ± 0.05 ^l^	11.85 ± 0.01 ^h^	4.04 ± 0.01 ^j^
08-21P X VS-8WAP	128.35 ± 0.23 ^a^	60.20 ± 0.01 ^a^	46.83 ± 0.04 ^a^	9.20 ± 0.03 ^a^
08-21P X TIS-12WAP	70.64 ± 0.04 ^j^	53.52 ± 0.20 ^c^	11.76 ± 0.04 ^h^	6.16 ± 0.03 ^c^
08-21P X TMS-16WAP	56.94 ± 0.23 ^l^	13.41 ± 0.23 ^m^	9.62 ± 0.06 ^k^	3.92 ± 0.02 ^k^
2019-11-2 X VS-8WAP	75.17 ± 0.19 ^g^	32.44 ± 0.10 ^g^	26.46 ± 0.27 ^d^	5.61 ± 0.04 ^f^
2019-11-2 X TIS-12WAP	71.14 ± 0.18 ^i^	30.76 ± 0.20 ^h^	7.98 ± 0.05 ^l^	4.99 ± 0.06 ^g^
2019-11-2 X TMS-16WAP	100.40 ± 0.08 ^b^	9.11 ± 0.00 ^n^	14.57 ± 0.30 ^g^	3.07 ± 0.08 ^l^
16-283P X VS-8WAP	82.66 ± 0.04 ^e^	41.42 ± 0.13 ^e^	40.50 ± 0.13 ^b^	6.00 ± 0.09 ^e^
16-283P X TIS-12WAP	71.89 ± 0.04 ^h^	17.19 ± 0.02 ^k^	10.88 ± 0.09 ^i^	4.99 ± 0.05 ^g^
16-283P X TMS-16WAP	86.53 ± 0.12 ^d^	33.16 ± 0.21 ^f^	18.44 ± 0.10 ^f^	4.70 ± 0.06 ^h^
2019-1-1 X VS-8WAP	88.12 ± 0.00 ^c^	22.21 ± 0.04 ^d^	22.21 ± 0.04 ^e^	6.07 ± 0.03 ^d^
2019-1-1 X TIS-12WAP	87.94 ± 0.23 ^c^	18.80 ± 24.42 ^j^	5.37 ± 0.01 ^n^	4.52 ± 0.00 ^i^
2019-1-1 X TMS-16WAP	52.43 ± 0.17 ^m^	9.11 ± 0.77 ^n^	18.80 ± 24.42 ^m^	3.07 ± 0.00 ^l^
LSD	2.88	2.43	2.14	0.71
Main and Interaction effects				
Genotype	10,075.27 ***	47,652.27 ***	23,659.72 ***	555.56 ^a^***
Harvest	228,192.57 ***	389,611.10 ***	276,844.88 ***	3467.32 ***
Genotype X harvesting stage	131,531.47 ***	44,032.25 ***	8265.98 ***	173.15 ***

Data are means and standard deviation (*n* = 3). Small letters within the same column represent the significant differences between genotypes and harvesting stages. *** represent significant levels at *p* < 0.001. VS-8WAP—vegetative stage 8 weeks after planting, TIS-12WAP—tuber initiation stage 12 weeks after planting, TMS-16WAP—tuber maturation stage 16 weeks after planting.

**Table 5:** FRAP, ABTS, and DPPH antioxidant activity of sweet potato leaves harvested during three harvesting stages.

Genotypes X Harvesting Stages	FRAP TEAC mM/g			ABTS IC_50_ mg/mL			DPPH IC_50_ mg/mL		
	S1	S2	Mean	S1	S2	Mean	S1	S2	Mean
Purple-purple X VS-8WAP	31.25±1.32 ^e^	48.75±0.87 ^bc^	40.00±9.64 ^d^	0.83±0.03 ^g^	0.42±0.03 ^de^	0.63±0.23 ^h^	2.22±0.09^f^	1.43±0.08 ^h^	1.83±0.44 ^h^
Purple-purple X TIS-12WAP	38.75±1.32 ^b^	49.92±0.76 ^b^	44.33±6.19 ^b^	0.55±0.02 ^cde^	0.36±0.01 ^bcd^	0.46±0.10 ^de^	1.46±0.06 ^d^	1.24±0.15 ^g^	1.35±0.16 ^f^
Purple-purple XTMS-16WAP	9.42±1.04 ^h^	39.42±1.04 ^f^	24.42±16.46 ^i^	1.31±0.11 ^h^	0.53±0.07 ^g^	0.92±0.43 ^i^	2.99±0.19 ^g^	1.85±0.06 ^i^	2.42±0.64 ^i^
08-21P X VS-8WAP	34.25±1.00 ^d^	46.58±1.16 ^d^	40.42±6.82 ^cd^	0.62±0.04 ^def^	0.36±0.00 ^bc^	0.49±0.14 ^ef^	1.54±0.01 ^de^	1.30±0.02 ^fg^	1.42±0.13 ^fg^
08-21P X TIS-12WAP	36.42±1.04 ^c^	46.92±2.75 ^cd^	41.67±6.05 ^c^	0.42±0.10 ^ab^	0.21±0.02 ^a^	0.32±0.13 ^b^	1.14±0.04 ^c^	0.68±0.04 ^c^	0.91±0.25 ^c^
08-21P X TMS-16WAP	7.75±0.01 ^h^	44.92±1.04 ^d^	26.33±20.37 ^h^	1.52±0.14 ^i^	0.62±0.05 ^h^	1.07±0.50 ^j^	4.33±0.18 ^h^	1.35±0.04 ^gh^	2.54±1.63 ^j^
2019-11-2 X VS-8WAP	40.08±1.44 ^b^	50.42±1.61 ^b^	45.25±5.82 ^b^	0.18±0.01 ^a^	0.22±0.01 ^a^	0.20±0.02 ^a^	0.73±0.061 ^a^	0.58±0.04 ^b^	0.66±0.10 ^a^
2019-11-2 X TIS-12WAP	50.08±1.61 ^a^	57.75±1.73 ^a^	53.92±4.46 ^a^	0.21±0.02 ^a^	0.23±0.01 ^a^	0.22±0.02 ^ab^	0.79±0.06 ^ab^	0.37±0.03 ^a^	0.58±0.23 ^a^
2019-11-2 X TMS-16WAP	29.58±0.29 ^e^	42.08±0.76 ^e^	35.83±6.87 ^e^	0.49±0.02 ^bc^	0.27±0.04 ^a^	0.38±0.12 ^c^	1.6±0.06 ^de^	0.71±0.07 ^c^	1.17±0.51 ^e^
16-283P X VS-8WAP	26.42±0.58 ^f^	35.75±0.50 ^g^	31.08±5.14 ^f^	0.64±0.09 ^ef^	0.41±0.02 ^cd^	0.52±0.14 ^fg^	2.17±0.11 ^f^	0.56±0.04 ^b^	1.37±0.88 ^f^
16-283P X TIS-12WAP	34.08±0.58 ^d^	40.08±2.02 ^ef^	37.08±3.55 ^e^	0.36±0.03 ^b^	0.34±0.02 ^b^	0.35±0.02 ^c^	1.17±0.01 ^c^	0.43±0.05 ^a^	0.80±0.41 ^b^
16-283P X TMS-16WAP	24.42±0.29 ^g^	33.92±0.76 ^g^	29.17±5.23 ^g^	0.54±0.02 ^cd^	0.47±0.04 ^ef^	0.50±0.05 ^gh^	1.54±0.14 ^de^	0.58±0.02 ^b^	1.06±0.53 ^d^
2019-1-1 X VS-8WAP	23.42±1.53 ^g^	23.25±0.50 ^i^	23.33±1.02 ^i^	1.63±0.03 ^j^	0.38±0.01 ^bcd^	1.01±0.69 ^i^	1.19±0.02 ^c^	1.06±0.07 ^e^	1.12±0.08 ^de^
2019-1-1 X TIS-12WAP	34.58±0.29 ^d^	28.92±0.29 ^h^	31.75±3.11 ^f^	0.67±0.06 ^f^	0.26±0.02 ^a^	0.47±0.23 ^cd^	0.93±0.04 ^b^	0.94±0.01 ^d^	0.94±0.02 ^c^
2019-1-1 X TMS-16WAP	9.25±0.50 ^h^	21.08±0.29 ^i^	15.17±6.49 ^j^	2.49±0.04 ^k^	0.52±0.06 ^fg^	1.31±1.08 ^k^	1.65±0.19 ^e^	1.28±0.02 ^fg^	1.47±0.23 ^g^
LSD	1.76	2.12	1.31	0.09	0.05	0.50	0.17	0.09	0.09

Data are means and standard deviation (*n* = 3). Small letters within the same column represent the significant differences between genotypes and harvesting stages. S1—season 1, S2—season 2, VS-8WAP—vegetative stage 8 weeks after planting, TIS-12WAP—tuber initiation stage 12 weeks after planting, TMS-16WAP—tuber maturation stage 16 weeks after planting.

## Data Availability

The original contributions presented in the study are included in the article/Supplementary Material, further inquiries can be directed to the corresponding author.

## Supplementary Materials

The following supporting information can be downloaded at: https://www.mdpi.com/article/10.3390/foods13111640/s1. Supplementary Figure S1: Harvested growth tips: A (Purple-purple), B (08-21P), C (2019-11-2), D (16-283P), and E (2019-1-1). Figure S2: Weather data during the growing season. The average temperatures (A) and average humidity (B). Supplementary Figure S3: Correlation between individual compound and the antioxidant power FRAP (A), ABTS scavenging activity (B), and DPPH scavenging activity (C); Supplementary Figures S4–S18: UPLC-QTOF/MS BPI chromatograms in ESI negative mode of different purple-fleshed sweet potato genotypes at different stages of harvesting. Supplementary Table S1: Soil nutrient status of plots during season 1 and season 2. Supplementary Table S2: Morphological characterisation of the above-ground mass of five purple-fleshed sweet potato genotypes according to the International sweet potato descriptors (CIP,1990). Supplementary Table S3: Main and interactions indicated by ANOVA on LAI, leaf chlorophyll, and leaf colour in five purple-fleshed genotypes during three different growth stages evaluated over two seasons. Supplementary Table S4: A comparison of LAI and chlorophyll in the leaves of different purple sweet potato genotypes at different stages of harvesting, including vegetative, tuber initiation, and tuber maturation. Supplementary Table S5A: The effect of different growth stages on L*, colour values in sweet potato leaf genotypes. Supplementary Table S5B: The effect of different growth stages on a* colour values in sweet potato leaf genotype. Supplementary Table S5C: The effect of different growth stages on b* colour values in sweet potato leaf genotypes. Table S6: Effects of genotype, harvesting stages, and their interaction on the level of total phenolic compounds (TPC), total carotenoids (TC), and the antioxidant activities in 5 purple-fleshed sweet potato leaves. Supplementary Table S7: Effects of genotype, harvesting stages, season, and their interaction on the accumulation of mineral content in five purple-fleshed genotype leaves harvested during three different growth stages.
