# Supplementary material for: Influence of Harvesting Stages on Phytonutrients and Antioxidant Properties of Leaves of Five Purple-Fleshed Sweet Potato (Ipomoea batatas) Genotypes
Source: Foods. 2024 May 24;13(11):1640. doi: 10.3390/foods13111640 (PMC11172356; doi:10.3390/foods13111640)
Supplement: Supplementary file 1 [file foods-13-01640-s001.zip › foods-2994217-supplementary.pdf]

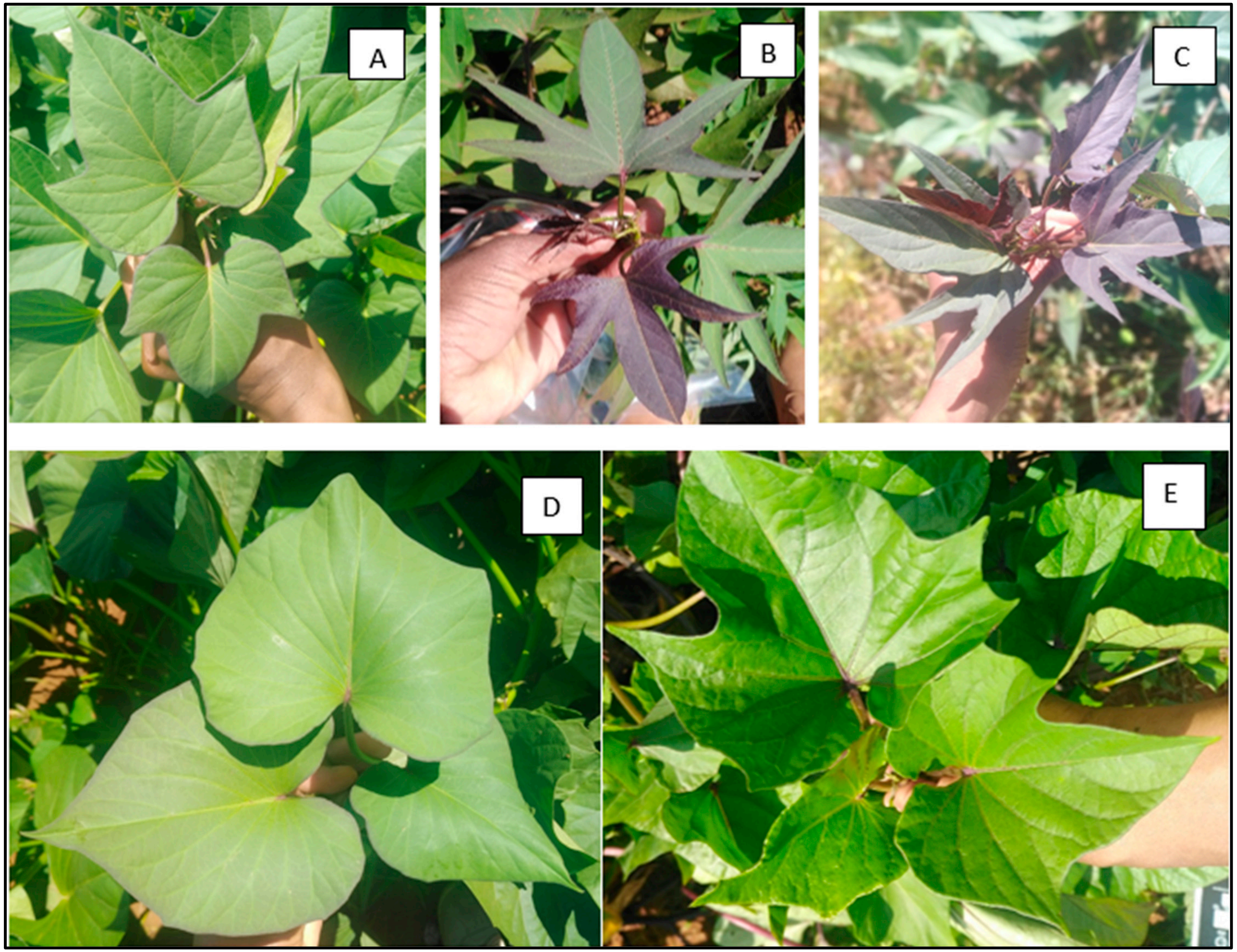

Supplementary Figure S1. Harvested growth tips: A (Purple-purple), B (08-21P), C (2019-11-2), D (16-283P), and E (2019-1-1)

Supplementary Table S1: Soil nutrient status of plots during season 1 and season 2.

	Sand particles (%) (2-0.5 mm)		Silt particles (%) (0.5-0.25 mm)		Clay particles (%) (0.25-0.106)
Land 41 N	72		6.0		22
Minerals mg/kg					
	P	K	Ca	Mg	Na
Plot 41 N	47	129	751	255	45.6
Plot 30	32.3	154	6.19	226	35.6

Supplementary Table S2. Morphological characterisation of the above-ground mass of five purple-fleshed sweet potato genotypes according to the International sweet potato descriptors (CIP,1990).

Genotypes	Plant type	Secondary vine pigmentation colour	Vine tip pubescence	Leaf outline	Leaf lobe type	Leaf lobe no	Abaxial leaf vein pigmentation	Mature foliage leaf colour	Immature foliage leaf colour	Petiole pigmentation
Purple-purple	Semi-erect	Green nodes	Moderate	Lobed	Slight	3	Green	Green	Green & green with purple edges	Green
08-21p	Semi-erect	Green tip	Sparse	Hastate	Deep	5-6	All veins partially purple	Green with purple veins on upper surface	Leaf 1-3 slightly purple, leaf 4-5 green	Totally/mostly purple

2019-11-2	Semi-erect	Purple tip	Heavy	Hastate	Moderate	3-5	Purple spots in several veins	Green	Purple both sides	Green with purple stripes
16-283p	Semi-erect	Purple nodes	Absent	Cordate	No lateral lobes	1	All veins mostly/totally purple	Green	Green & green with purple edges	Green with purple near leaf
2019-1-1	Semi-erect	Purple base	Sparse	Lobed	Very slight teeth	5-9	Lower surface and veins totally purple	Green with purple veins on upper surface	Green & green with purple vein on upper surface	Totally/mostly purple

Supplementary Table S3. Main and interactions indicated by ANOVA on LAI, leaf chlorophyll, and leaf colour in five purple-fleshed genotypes during three different growth stages evaluated over two seasons

	LAI	Chlorophyll	L*	a*	b*	L*below	a*below	b*below
Genotype	0.23^{ns}	22.12**	87.66***	253.34***	221.90***	123.69***	236.71***	87.75***
Harvesting stage	17.52***	8.98^{ns}	223.74***	10.16*	51.23***	257.19***	2.30^{ns}	34.52***
Season	0.71^{ns}	98.73***	8958.53***	238.83***	1341.89***	12448.74***	127.19***	1545.11***
Genotype X harvesting stage	0.23*	7.31*	30.58***	6.72*	13.55***	13.83^{ns}	4.12^{ns}	5.89**
Genotype x season	0.03^{ns}	40.41***	40.70***	52.51***	56.00***	62.30***	46.35***	26.92***
Harvesting stage X season	2.11***	18.70*	60.69***	39.44***	14.60***	133.09***	14.49*	8.55**
Genotype x Harvesting stage x Season	0.12^{ns}	19.49***	22.12***	5.44^{ns}	5.45**	31.29**	2.01^{ns}	1.71^{ns}

***, **, * and ns represent significant level at p <0.001, p<0.01, p<0.05 and no significant respectively.

Supplementary Table S4. A comparison of LAI and chlorophyll in the leaves of different purple sweet potato genotypes at different stages of harvesting, including vegetative, tuber initiation, and tuber maturation

Genotypes	Leaf area index			Chlorophyll		
Harvesting stages	Season 1	Season 2	Mean	Season 1	Season 2	Mean
Purple-purple X VS-8WAP	1.59±0.49 ab	1.52±0.16 b	1,55±0.33 b	42.87±1.05 de	44.46±2.62 cde	43,66±1.96 d
Purple-purple X TIS-12WAP	0.87±0.60 c	0.47±0.07 c	0,67±0.44 c	46.83±2.04 abc	42.43±1.74 defg	44,63±2.95 bcd
Purple-purple X TMS-16WAP	0.31±0.29 d	0.04±0.03 d	0,17±0.24 d	47.85±1.68 abc	42.02±0.86 efg	44,94±3.41 bcd
08-21p X VS-8WAP	1.44±0.14 b	1.69±0.31 b	1,57±0.26 b	49.05±0.42 ab	44.08±1.30 defg	46,57±2.86 ab
08-21p X TIS-12WAP	1.19±0.29 bc	0.30±0.26 cd	0,75±0.55 c	45.72±1.21 bcd	44.40±2.64 cdef	45,06±1.97 bcd
08-21p X TMS-16WAP	0.12±0.09 d	0.05±0.03 d	0,08±0.07 d	45.19±0.63 cd	41.75±1.41 eg	43,47±2.12 d
2019-11-2 X VS-8WAP	1.15±0.43 bc	1.70±0.65 b	1,43±0.58 b	48.00±2.05 abc	43.03±0.29 defg	45,52±3.02 bcd
2019-11-2 X TIS-12WAP	1.27±0.29 bc	0.28±0.10 cd	0,77±0.58 c	49.16±0.67 a	43.43±0.94 defg	46,30±3.22 abc
2019-11-2 X TMS-16WAP	0.31±0.25 d	0.13±0.12 cd	0,22±0.20 d	47.49±2.02 abc	41.20±2.50 g	44,35±4.00 cd
16-283p X VS-8WAP	2.11±0.16 a	2.41±0.33 a	2,26±0.29 a	42.99±4.17 de	47.09±1.75 abc	45,04±3.64 bcd
16-283p X TIS-12WAP	1.08±0.53 bc	0.43±0.05 cd	0,75±0.49 c	48.87±0.99 ab	44.10±1.09 defg	46,49±2.77 ab
16-283p X TMS-16WAP	0.05±0.04 d	0.31±0.04 cd	0,18±0.31 d	39.62±0.81 e	47.50±1.49 ab	43,56±4.45 d
2019-1-1 X VS-8WAP	1.51±0.23 b	1.70±0.08 b	1,60±0.19 b	47.53±1.54 abc	47.79±1.68 a	47,66±1.45 a
2019-1-1 X TIS-12WAP	1.27±0.04 bc	0.34±0.12 cd	0,81±0.52 c	48.08±1.29 abc	45.03±0.94 bcd	46,56±2.04 ab
2019-1-1 X TMS-16WAP	0.06±0.01 d	0.29±0.07 cd	0,18±0.13 d	47.97±3.79 abc	47.48±2.06 ab	47,73±2.74 a
**LSD**	0.54	0.41	0.33	3.30	2.97	2.11

Data are means and standard deviation (n=3). Small letters within the same column represent the significant differences between genotypes and harvesting stages. S1~season 1, S2~season 2, VS-8WAP~vegetative stage 8 weeks after planting, TIS-12WAP~tuber initiation stage 12 weeks after planting, TMS-

16WAP~tuber maturation stage 16 weeks after planting. Means followed by the same letter within the row are not significantly different ($p < 0.05$), each of the samples was replicated three times, and the results are expressed as mean ± standard deviation.

Supplementary Table S5A. The effect of different growth stages on *L, colour values in sweet potato leaf genotypes**

*L** value						
Genotype x harvesting stage	Adaxial leaf side			Abaxial leaf side		
	S1	S2	Mean	S1	S2	Mean
Purple-purple X VS-8WAP	6.67±1.158 f	28.83±1.442 fg	17.75±12.198 d	11.05±1.076 f	45.53±2.380 abc	28.29±18.957 cde
Purple-purple X TIS-12WAP	12.64±1.327 e	36.84±3.297 abc	24.74±13.445 bc	18.44±3.039 de	47.00±3.183 ab	32.72±15.889 bc
Purple-purple X TMS-16WAP	16.81±1.347 bc	38.36±0.739 ab	27.59±11.842 a	26.88±2.769 a	47.53±4.170 ab	37.21±11.742 a
08-21P X VS-8WAP	13.92±0.461 de	35.30±2.334 abcd	24.61±11.808 bc	20.95±1.182 bcde	42.37±1.52 bcd	31.66±11.796 bcd
08-21P X TIS-12WAP	17.42±1.226 ab	37.07±2.311 abc	27.24±10.886 a	25.39±1.319 ab	49.94±1.042 a	37.66±13.493 a
08-21P X TMS-16WAP	19.89±0.583 a	34.21±0.993 bcd	27.05±7.878 a	26.30±2.981 a	48.05±2.966 ab	37.17±12.210 a
2019-11-2 X VS-8WAP	7.54±1.400 f	28.67±3.028 fg	18.10±11.761 d	17.98±6.499 e	39.24±7.094 cd	28.61±13.141 bcde
2019-11-2 X TIS-12WAP	12.43±1.741 e	26.25±1.788 g	19.34±7.729 d	20.80±0.981 bcde	37.88±3.849 d	29.34±9.684 bcde
2019-11-2 X TMS-16WAP	14.09±1.661 de	34.30±1.069 bcd	24.20±11.141 bc	20.69±2.988 bcde	42.83±2.176 bcd	31.76±12.349 bcd
16-283P X VS-8WAP	8.10±0.935 f	27.90±3.236 fg	18.00±11.052 d	11.58±2.403 f	39.62±5.125 cd	25.60±15.767 e
16-283P X TIS-12WAP	15.43±1.128 bcd	29.74±0.143 efg	22.58±7.872 c	20.11±1.160 cde	37.53±3.730 d	28.82±9.858 bcde
16-283P X TMS-16WAP	17.56±1.050 ab	33.46±2.367 cde	25.51±8.865 ab	24.72±0.994 abc	39.31±0.381 cd	32.02±8.024 bcd
2019-1-1 X VS-8WAP	7.51±0.961 f	38.35±2.240 ab	22.93±16.965 c	10.50±1.154 f	45.01±2.903 abc	27.75±19.004 de
2019-1-1 X TIS-12WAP	14.77±2.226 cde	39.37±1.621 a	27.07±13.586 a	19.04±1.687 de	46.77±2.106 ab	32.90±15.286 b
2019-1-1 X TMS-16WAP	15.61±2.234 bcd	31.0±4.413 def	23.33±9.011 bc	23.10±2.057 abcd	41.73±1.729 bcd	32.42±10.344 bc
Lsd	2.39	3.95		4.39	5.77	

Supplementary Table S5B. The effect of different growth stages on *a colour values in sweet potato leaf genotype**

*a** value						
Genotype x harvesting stage	Adaxial leaf side			Abaxial leaf side		
	S1	S2	Mean	S1	S2	Mean
Purple-purple X VS-8WAP	-4.134±0.786 b	-11.951±0.712 cd	-8.042±4.334 bcd	-4.826±0.326 cde	-11.503±0.923 cd	-8.164±3.71 bcde
Purple-purple X TIS-12WAP	-6.508±0.517 c	-13.721±0.599 d	-10.115±3.983 de	-6.244±0.721 fg	-13.353±1.084 d	-9.799±3.980 e
Purple-purple X TMS-16WAP	-7.302±0.889 cd	-12.651±0.368 cd	-9.976±2.992 de	-7.732±0.789 h	-11.340±1.046 cd	-9.536±2.143 de
08-21P X VS-8WAP	-6.703±0.420 c	-12.344±2.470 cd	-9.524±3.472 cde	-5.640±0.622 def	-9.683±1.000 bcd	-7.661±2.34 bcd
08-21P X TIS-12WAP	-6.753±0.920 c	-9.843±3.070 cd	-8.298±2.641 bcde	-5.816±1.312 defg	-9.002±1.460 bc	-7.409±2.14 bc
08-21P X TMS-16WAP	-8.464±0.239 d	-5.470±1.628 b	-6.967±1.942 b	-6.114±0.697 efg	-5.985±1.339 b	-6.049±0.96 b
2019-11-2 X VS-8WAP	-1.943±0.766 a	-0.033±1.688 a	-0.988±1.571 a	-2.260±0.634 b	-1.165±2.135 a	-1.713±1.531 a
2019-11-2 X TIS-12WAP	-1.573±0.852 a	0.458±0.842 a	-0.557±1.346 a	-0.907±0.407 a	1.071±0.233 a	0.082±1.124 a
2019-11-2 X TMS-16WAP	-1.166±1.341 a	0.449±1.126 a	-0.358±1.417 a	-0.948±1.386 a	2.306±3.178 a	0.679±2.826 a
16-283P X VS-8WAP	-4.798±0.455 b	-10.109±0.409 cd	-7.454±2.934 bc	-4.319±0.826 c	-9.091±1.212 bc	-6.705±2.773 bc
16-283P X TIS-12WAP	-7.879±0.502 cd	-8.827±0.360 cd	-8.353±0.650 de	-6.356±0.392 fg	-7.616±2.810 bc	-6.986±1.922 bc
16-283P X TMS-16WAP	-7.890±0.727 cd	-9.170±1.754 c	-8.530±1.391 bcde	-7.117±0.247 gh	-8.578±1.409 bc	-7.848±1.208 bcde
2019-1-1 X VS-8WAP	-4.624±0.988 b	-14.087±0.417 d	-9.355±5.227 cde	-4.646±0.243 cd	-9.524±2.610 bc	-7.085±3.144 bc
2019-1-1 X TIS-12WAP	-7.645±0.847 c	-13.559±0.547 d	-10.602±3.302 e	-6.417±0.469 fg	-11.268±0.552 cd	-8.843±2.696 cde
2019-1-1 X TMS-16WAP	-7.505±0.293 cd	-9.278±1.255 bc	-8.392±1.267 bcde	-7.031±0.353 gh	-9.364±0.606 bc	-8.198±1.353 bcde
**Lsd**	1.28	3.69		1.21	3.4	

Supplementary Table S5C. The effect of different growth stages on *b colour values in sweet potato leaf genotypes**

*b** value						
Genotype x harvesting stage	Adaxial leaf side			Abaxial leaf side		
	S1	S2	Mean	S1	S2	Mean
Purple-purple X VS-8WAP	5.43±1.033 def	17.73±1.719 bc	11.58±6.857 fg	7.71±0.646 d	20.41±1.030 bc	14.06±7.001 cd
Purple-purple X TIS-12WAP	9.69±0.816 c	23.22±0.995 a	16.46±7.454 ab	10.63±0.611 bc	23.47±0.786 a	17.05±7.062 a
Purple-purple X TMS-16WAP	11.13±1.407 abc	22.17±0.803 a	16.65±6.130 a	12.95±1.487 a	21.89±1.549 ab	17.42±5.080 a
08-21P X VS-8WAP	9.68±0.313 c	17.92±2.117 bc	13.80±4.712 cde	10.10±0.661 c	17.75±0.327 cd	13.92±4.217 cd
08-21P X TIS-12WAP	9.48±0.421 c	17.11±2.401 bc	13.29±4.453 e	10.66±0.486 bc	18.25±2.153 cd	14.46±4.384 cd
08-21P X TMS-16WAP	12.33±0.438 a	14.58±0.830 d	13.46±1.366 de	10.89±0.800 bc	17.80±1.163 cd	14.34±3.885 cd
2019-11-2 X VS-8WAP	4.37±1.159 f	8.39±1.268 ef	6.38±2.458 hi	7.61±0.654 d	13.75±3.682 e	10.68±4.111 fg
2019-11-2 X TIS-12WAP	4.81±0.820 ef	6.30±0.492 f	5.55±1.016 i	7.93±0.289 d	11.63±0.811 e	9.78±2.099 g
2019-11-2 X TMS-16WAP	5.21±1.165 def	9.70±1.977 e	7.45±2.858 h	8.30±1.050 d	13.56±2.183 e	10.93±3.259 fg
16-283P X VS-8WAP	6.56±0.963 d	14.59±0.298 d	10.57±4.442 g	7.72±1.679 d	16.51±1.607 d	12.11±5.034 ef
16-283P X TIS-12WAP	10.33±0.868 bc	16.14±1.849 cd	13.24±3.436 e	10.59±0.188 bc	18.49±1.337 cd	14.54±4.410 cd
16-283P X TMS-16WAP	11.56±0.867 ab	18.07±1.564 bc	14.81±3.738 cd	11.80±0.420 ab	18.59±0.952 cd	15.19±3.777 bc
2019-1-1 X VS-8WAP	6.45±1.138 de	19.40±0.454 b	12.92±7.132 ef	7.82±0.626 d	19.01±0.899 bcd	13.41±6.167 de
2019-1-1 X TIS-12WAP	11.66±1.193 ab	21.91±0.269 a	16.78±5.667 a	11.68±0.481 ab	21.64±0.635 ab	16.66±5.480 ab
2019-1-1 X TMS-16WAP	11.47±0.364 ab	18.78±1.036 b	15.13±4.062 bc	12.36±0.105 a	20.31±1.252 bc	16.34±4.428 ab
Lsd	1.55	2.31		1.35	2.69	

Data are means and standard deviation (n=3). Small letters within the same column represent the significant differences between genotypes and harvesting stages. S1~season 1, S2~season 2, VS-8WAP~vegetative stage 8 weeks after planting, TIS-12WAP~tuber initiation stage 12 weeks after planting, TMS-16WAP~tuber maturation stage 16 weeks after planting

Supplementary Table S6. Effects of genotype, harvesting stages, and their interaction on the level of total phenolic compounds (TPC), total carotenoids (TC), and the antioxidant activities in 5 purple-fleshed sweet potato leaves.

Main and interaction effect	TPC	TC	FRAP	ABTS	DPPH
Genotype	18123.84***	1137.97***	1010.85***	339.62***	3,64***
Harvest stage	75251.33***	3770.02***	1998.19***	678.09***	5.81***
Season	94818.91***	1986.59***	3240.12***	2447.29***	12.35***
Genotype x harvest stage	2386.43***	215.54***	66,43***	74.14***	0,86***
Genotype x season	1565.60***	117.72***	266.88***	501.65***	0,86***
Harvesting stage x season	4624.58***	301.92***	408.27***	387.61***	1.67***
Genotype x Harvesting stage x Season	2753.60***	45.2***	49.89***	74.71***	0.67***

*** represent significant level at p <0.001.

Supplementary Table S7: Effects of genotype, harvesting stages, season, and their interaction on the accumulation of mineral content in five purple-fleshed genotype leaves harvested during three different growth stages.

	**N**	**P**	**K**	**Ca**	**Mg**	**Fe**	**Cu**	**Zn**	**Mn**	**B**
**Genotype**	1.521E+04***	0.05***	5718.617***	2444.32***	0.01***	1.600E+05***	4590.67***	13478.66***	4.549E+04***	4.229E+03***
**Harvest stage**	1.460E+05***	0.27**	35692.300***	1075.43***	0.045***	9.527E+04***	58008.46***	347893.70***	1.907E+04***	1.544E+05***
**Genotype X harvest stage**	2.850E+03***	0.01***	1612.736***	119.51***	0.005***	4.862E+04***	1818.854***	16015.40***	1.828E+03***	5.165E+04***
**Season**	3.097E+02***	0.08***	1344.631***	5775.48***	0.114***	1.310E+05***	8.503**	1718.61***	1.921E+03***	2.474E+03***
**Genotype x season**	2.008E+02***	0.001***	54.221***	150.06***	0.004***	2.175E+03***	37.350***	12.80***	1.668E+03***	2.351E+02***

**Harvesting stage X season**	1.270E+03***	0.02***	248.511***	302.50***	0.005***	2.035E+02***	9.93***	163.47***	1.483E+03***	2.762E+04***
**Genotype x Harvesting stage x Season**	6.947E+01***	0.01***	15.525***	111.68***	0.003***	1.115E+03***	75.19***	59.92***	1.042E+03***	9.023E+0.1***

*** represent significant level at p <0.001.

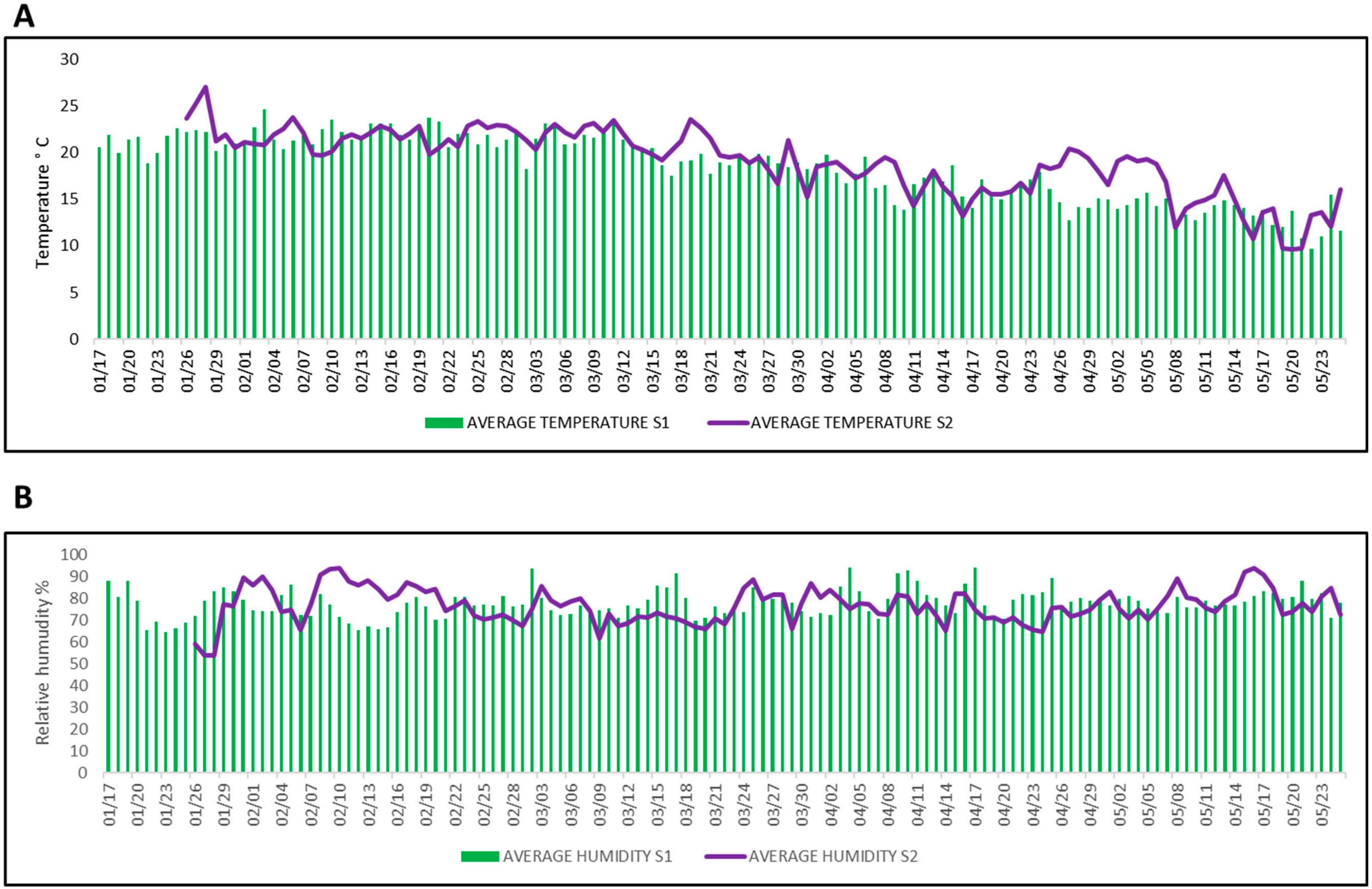

Supplementary Figure S2. Weather data during the growing season. The average temperatures (A) and average humidity (B).

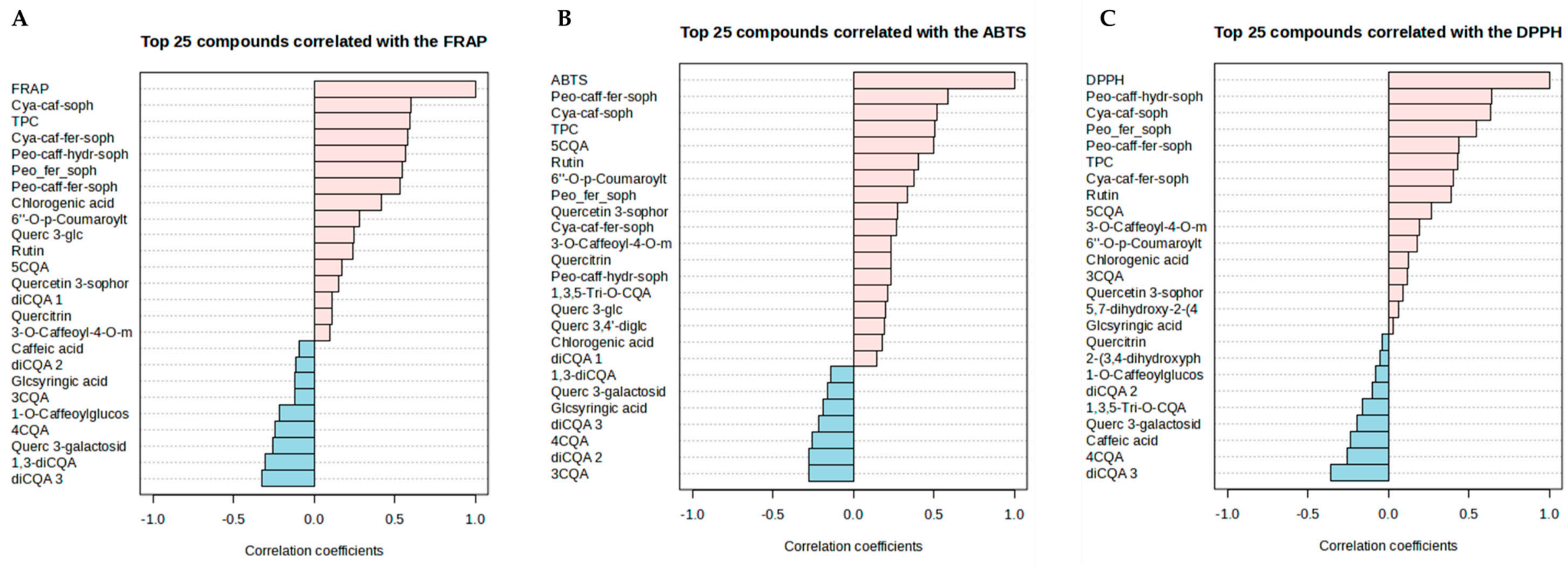

Supplementary Figure S3. Correlation between individual compound and the antioxidant power FRAP (A), ABTS scavenging activity (B), and DPPH scavenging activity (C)

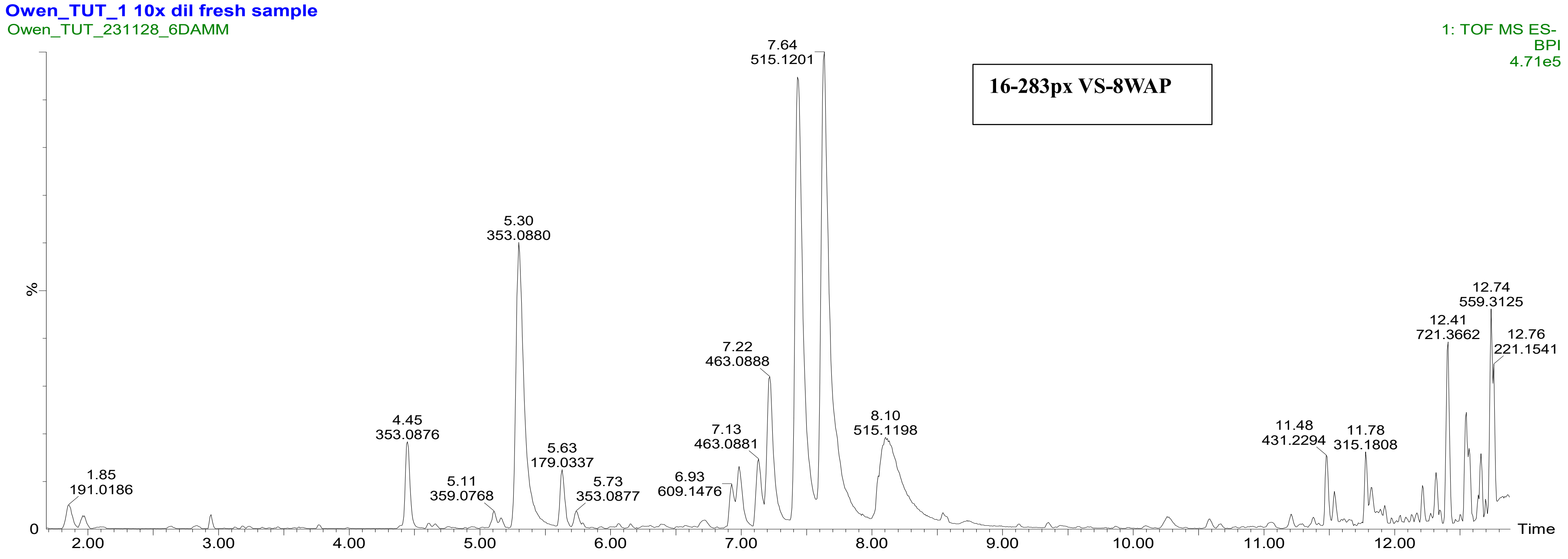

Owen_TUT_1 10x dil fresh sample
Owen_TUT_231128_6DAMM
1: TOF MS ES-
BPI
4.71e5
16-283px VS-8WAP
%
0
Time
2.00
3.00
4.00
5.00
6.00
7.00
8.00
9.00
10.00
11.00
12.00
1.85
191.0186
4.45
353.0876
5.11
359.0768
5.30
353.0880
5.63
179.0337
5.73
353.0877
6.93
609.1476
7.13
463.0881
7.22
463.0888
7.64
515.1201
8.10
515.1198
11.48
431.2294
11.78
315.1808
12.41
721.3662
12.74
559.3125
12.76
221.1541

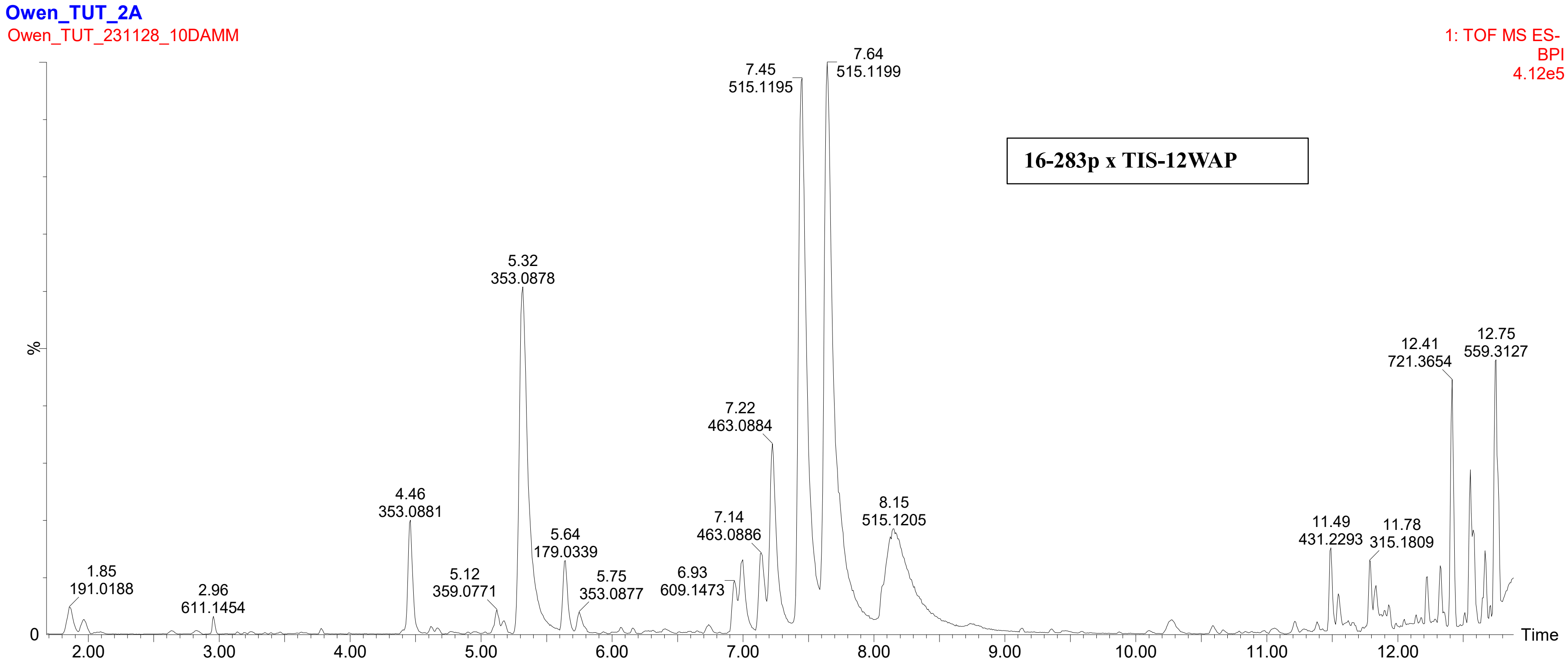

Owen_TUT_2A
Owen_TUT_231128_10DAMM
1: TOF MS ES-
BPI
4.12e5
16-283p x TIS-12WAP
%
0
Time
1.85 191.0188
2.96 611.1454
4.46 353.0881
5.12 359.0771
5.32 353.0878
5.64 179.0339
5.75 353.0877
6.93 609.1473
7.14 463.0886
7.22 463.0884
7.45 515.1195
7.64 515.1199
8.15 515.1205
11.49 431.2293
11.78 315.1809
12.41 721.3654
12.75 559.3127
2.00
3.00
4.00
5.00
6.00
7.00
8.00
9.00
10.00
11.00
12.00

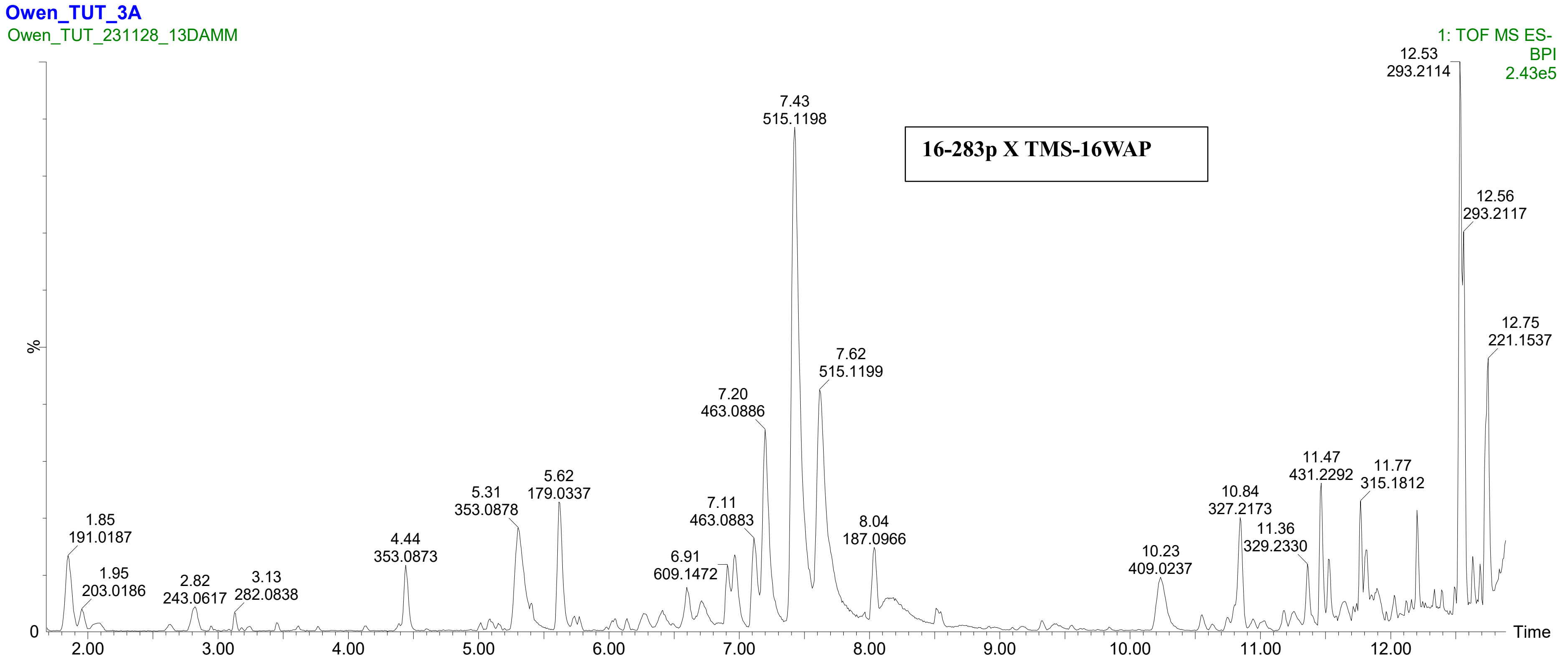

Owen_TUT_3A
Owen_TUT_231128_13DAMM
1: TOF MS ES-
BPI
2.43e5
16-283p X TMS-16WAP
12.53
293.2114
7.43
515.1198
12.56
293.2117
12.75
221.1537
7.62
515.1199
7.20
463.0886
11.47
431.2292
11.77
315.1812
5.62
179.0337
5.31
353.0878
10.84
327.2173
7.11
463.0883
8.04
187.0966
11.36
329.2330
1.85
191.0187
4.44
353.0873
6.91
609.1472
10.23
409.0237
1.95
203.0186
2.82
243.0617
3.13
282.0838
%
0
Time
2.00
3.00
4.00
5.00
6.00
7.00
8.00
9.00
10.00
11.00
12.00

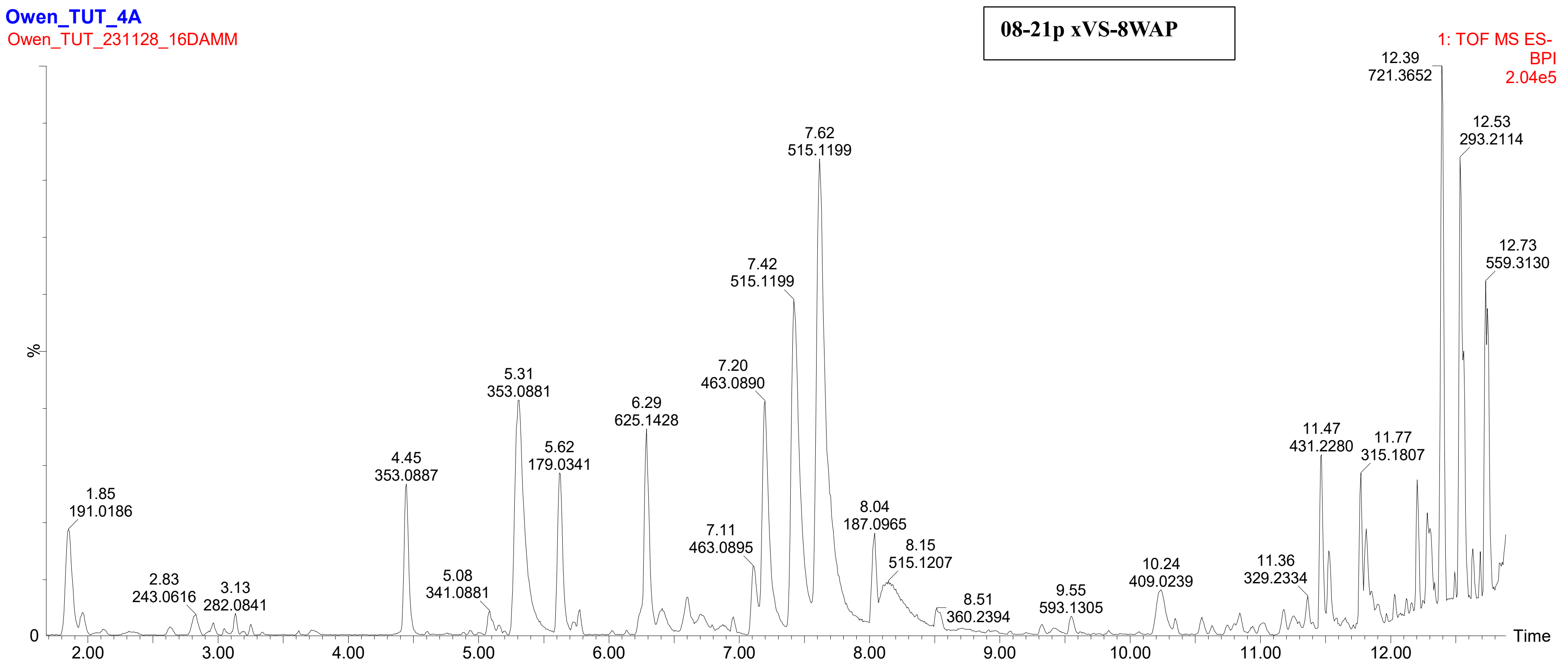
Owen_TUT_4A
Owen_TUT_231128_16DAMM
08-21p xVS-8WAP
1: TOF MS ES-
BPI
2.04e5
%
0
Time
1.85
191.0186
2.83
243.0616
3.13
282.0841
4.45
353.0887
5.08
341.0881
5.31
353.0881
5.62
179.0341
6.29
625.1428
7.11
463.0895
7.20
463.0890
7.42
515.1199
7.62
515.1199
8.04
187.0965
8.15
515.1207
8.51
360.2394
9.55
593.1305
10.24
409.0239
11.36
329.2334
11.47
431.2280
11.77
315.1807
12.39
721.3652
12.53
293.2114
12.73
559.3130
2.00
3.00
4.00
5.00
6.00
7.00
8.00
9.00
10.00
11.00
12.00

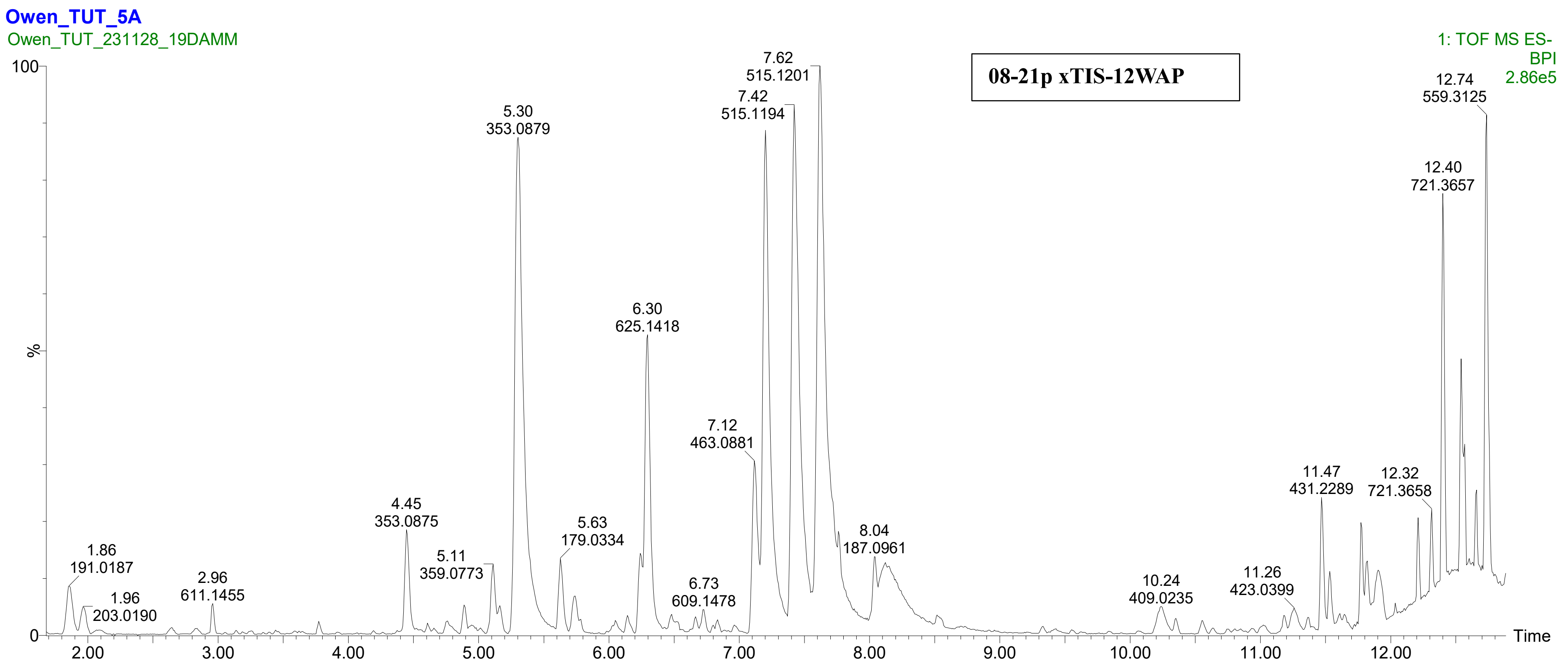
Owen_TUT_5A
Owen_TUT_231128_19DAMM
08-21p xTIS-12WAP
1: TOF MS ES-
BPI
2.86e5
100
%
0
Time
1.86
191.0187
1.96
203.0190
2.96
611.1455
4.45
353.0875
5.11
359.0773
5.30
353.0879
5.63
179.0334
6.30
625.1418
6.73
609.1478
7.12
463.0881
7.42
515.1194
7.62
515.1201
8.04
187.0961
10.24
409.0235
11.26
423.0399
11.47
431.2289
12.32
721.3658
12.40
721.3657
12.74
559.3125
2.00
3.00
4.00
5.00
6.00
7.00
8.00
9.00
10.00
11.00
12.00

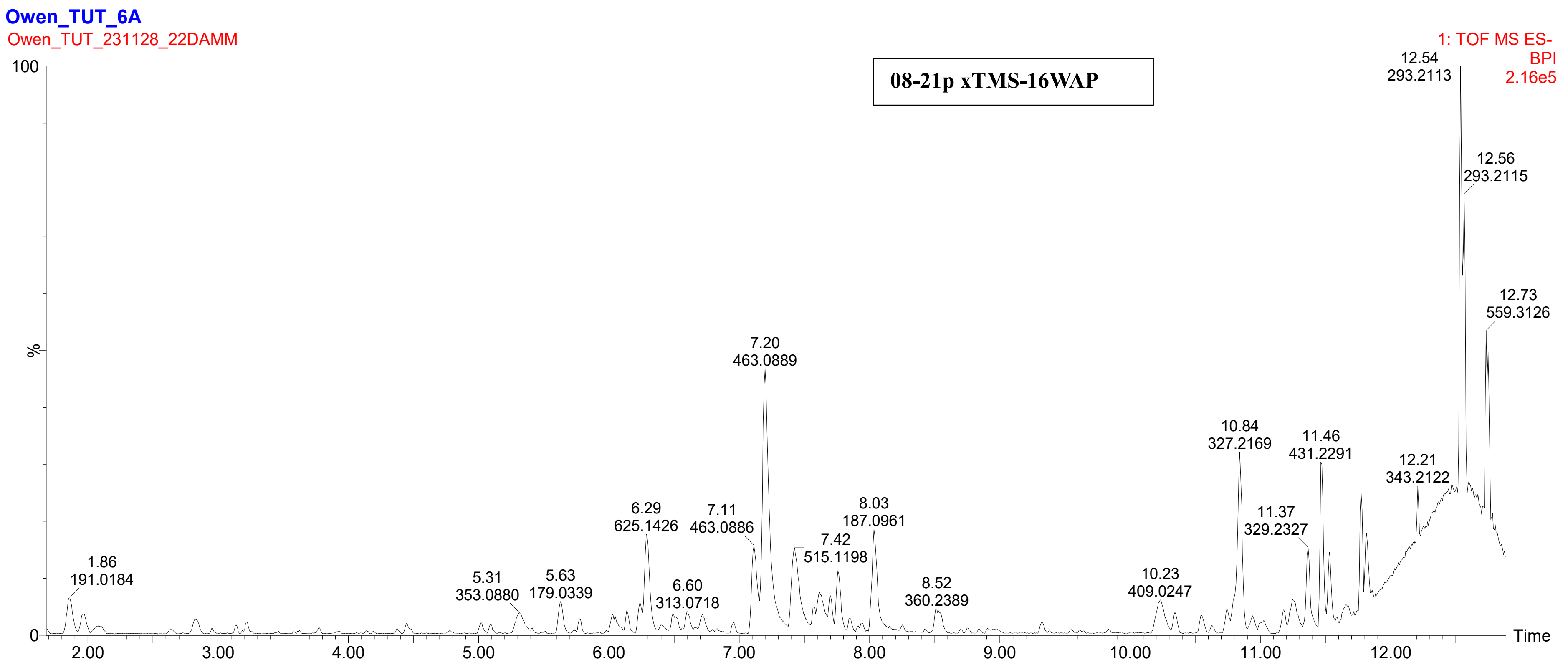
Owen_TUT_6A
Owen_TUT_231128_22DAMM
08-21p xTMS-16WAP
1: TOF MS ES-
BPI
2.16e5
100
%
0
12.54
293.2113
12.56
293.2115
12.73
559.3126
7.20
463.0889
10.84
327.2169
11.46
431.2291
12.21
343.2122
6.29
625.1426
7.11
463.0886
8.03
187.0961
11.37
329.2327
7.42
515.1198
1.86
191.0184
5.31
353.0880
5.63
179.0339
6.60
313.0718
8.52
360.2389
10.23
409.0247
2.00
3.00
4.00
5.00
6.00
7.00
8.00
9.00
10.00
11.00
12.00
Time

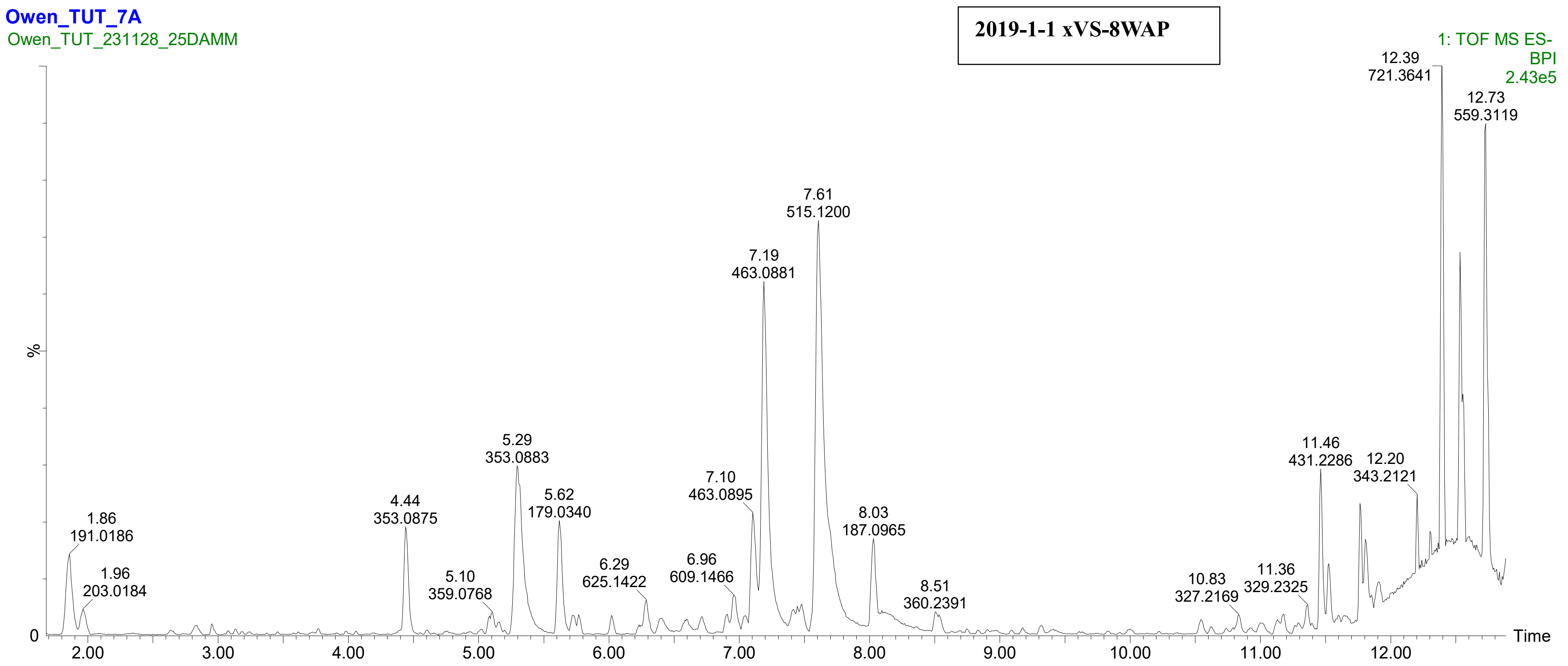
Owen_TUT_7A
Owen_TUT_231128_25DAMM
2019-1-1 xVS-8WAP
1: TOF MS ES-
BPI
2.43e5
12.39
721.3641
12.73
559.3119
7.61
515.1200
7.19
463.0881
%
5.29
353.0883
11.46
431.2286
12.20
343.2121
7.10
463.0895
4.44
353.0875
5.62
179.0340
8.03
187.0965
1.86
191.0186
6.29
625.1422
6.96
609.1466
1.96
203.0184
5.10
359.0768
8.51
360.2391
10.83
327.2169
11.36
329.2325
0
2.00
3.00
4.00
5.00
6.00
7.00
8.00
9.00
10.00
11.00
12.00
Time

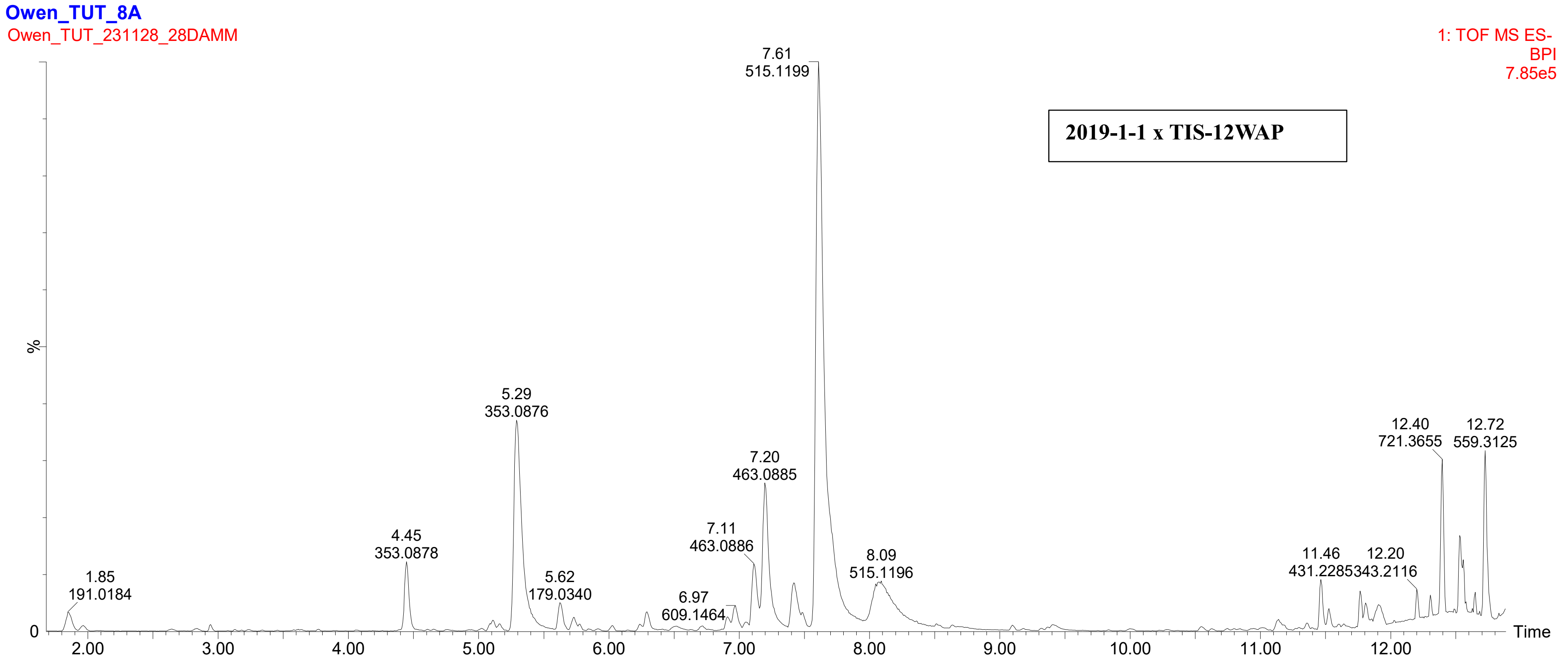
Owen_TUT_8A
Owen_TUT_231128_28DAMM
1: TOF MS ES-
BPI
7.85e5
2019-1-1 x TIS-12WAP
%
0
Time
1.85
191.0184
4.45
353.0878
5.29
353.0876
5.62
179.0340
6.97
609.1464
7.11
463.0886
7.20
463.0885
7.61
515.1199
8.09
515.1196
11.46
431.2285
12.20
343.2116
12.40
721.3655
12.72
559.3125
2.00
3.00
4.00
5.00
6.00
7.00
8.00
9.00
10.00
11.00
12.00

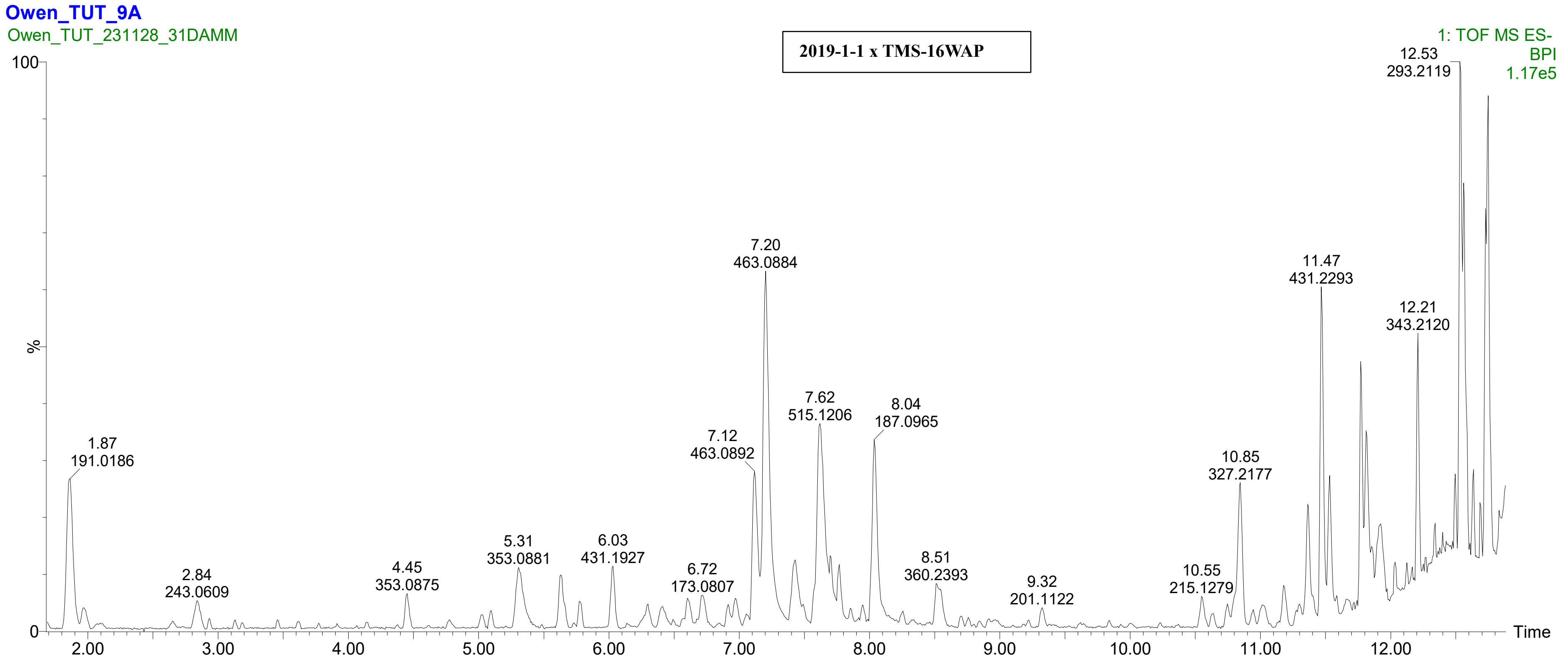
Owen_TUT_9A
Owen_TUT_231128_31DAMM
2019-1-1 x TMS-16WAP
1: TOF MS ES-
BPI
1.17e5
100
%
0
12.53
293.2119
7.20
463.0884
11.47
431.2293
12.21
343.2120
7.62
515.1206
8.04
187.0965
7.12
463.0892
1.87
191.0186
10.85
327.2177
5.31
353.0881
6.03
431.1927
6.72
173.0807
8.51
360.2393
4.45
353.0875
2.84
243.0609
9.32
201.1122
10.55
215.1279
2.00
3.00
4.00
5.00
6.00
7.00
8.00
9.00
10.00
11.00
12.00
Time

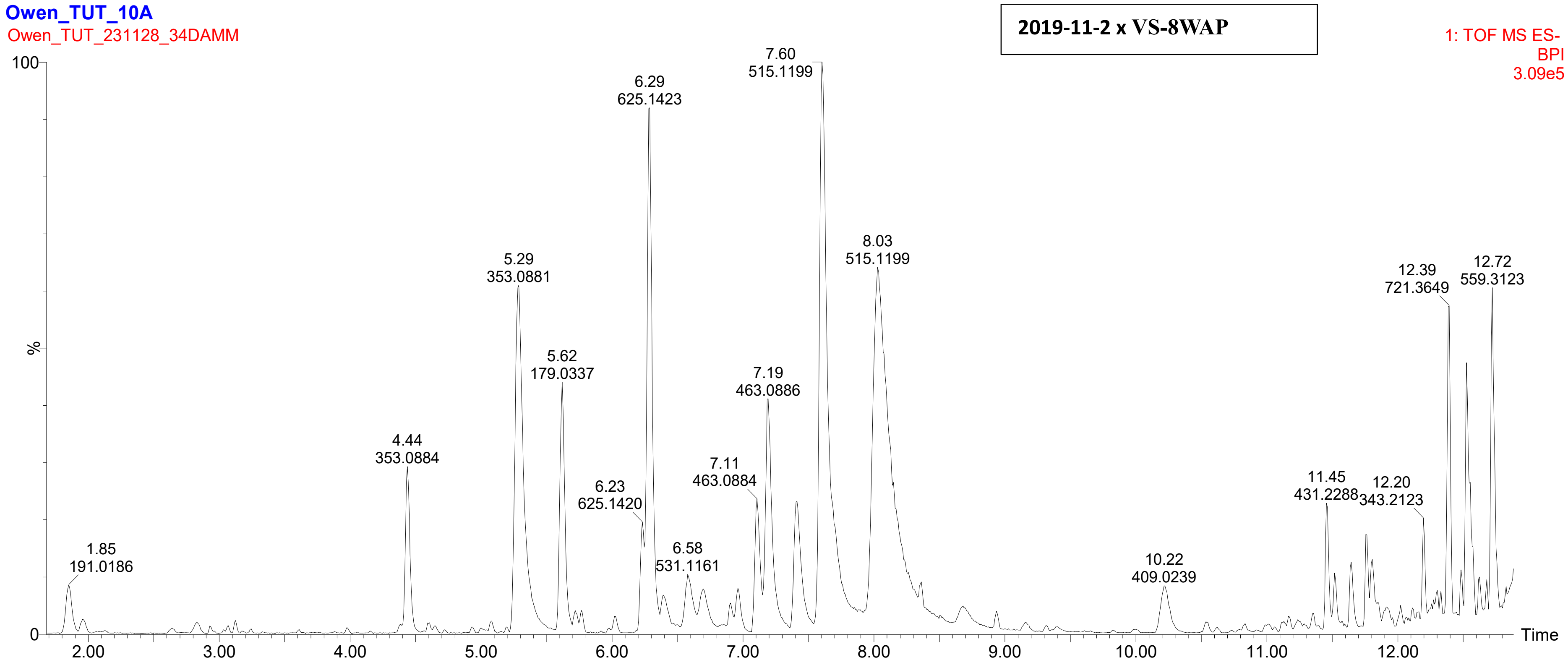
Owen_TUT_10A
Owen_TUT_231128_34DAMM
2019-11-2 x VS-8WAP
1: TOF MS ES-
BPI
3.09e5
100
%
0
7.60
515.1199
6.29
625.1423
8.03
515.1199
5.29
353.0881
12.39
721.3649
12.72
559.3123
5.62
179.0337
7.19
463.0886
4.44
353.0884
7.11
463.0884
6.23
625.1420
11.45
431.2288
12.20
343.2123
6.58
531.1161
1.85
191.0186
10.22
409.0239
2.00
3.00
4.00
5.00
6.00
7.00
8.00
9.00
10.00
11.00
12.00
Time

Owen_TUT_11A

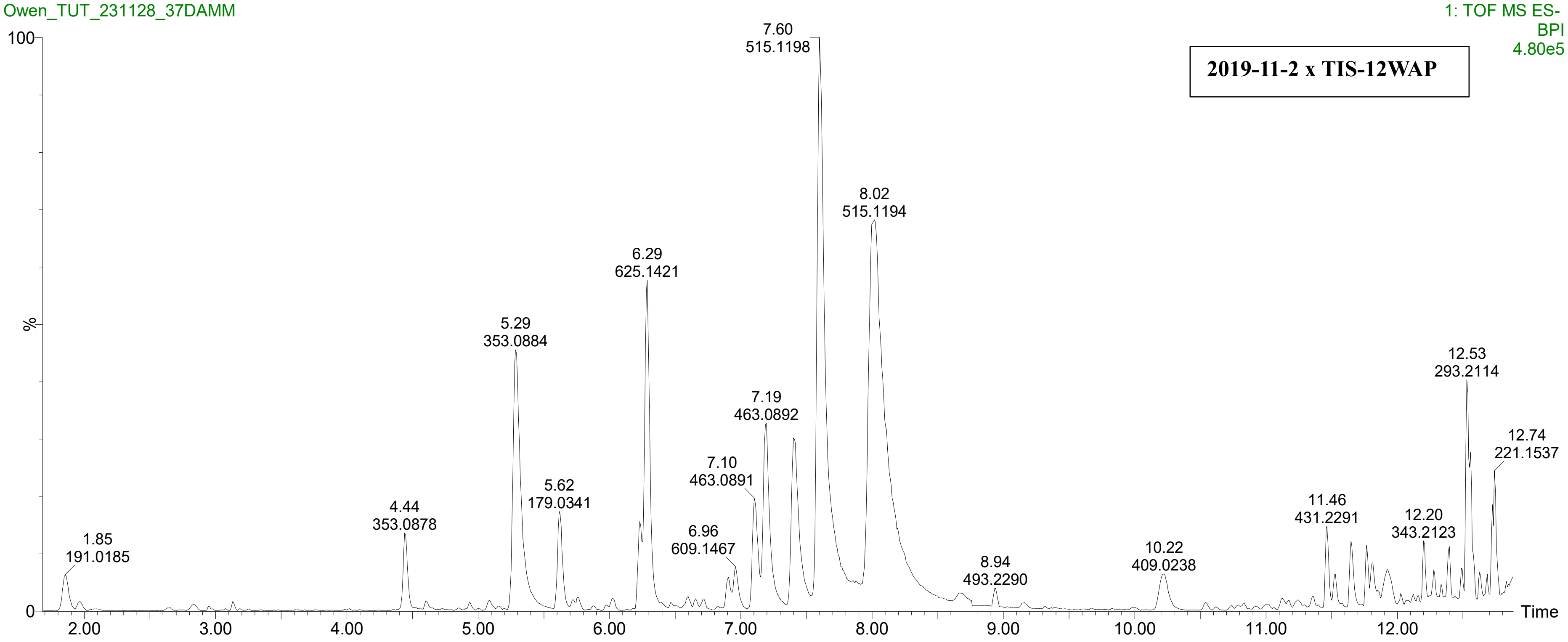
Owen_TUT_231128_37DAMM
1: TOF MS ES-
BPI
4.80e5
2019-11-2 x TIS-12WAP
100
%
0
1.85
191.0185
4.44
353.0878
5.29
353.0884
5.62
179.0341
6.29
625.1421
6.96
609.1467
7.10
463.0891
7.19
463.0892
7.60
515.1198
8.02
515.1194
8.94
493.2290
10.22
409.0238
11.46
431.2291
12.20
343.2123
12.53
293.2114
12.74
221.1537
2.00
3.00
4.00
5.00
6.00
7.00
8.00
9.00
10.00
11.00
12.00
Time

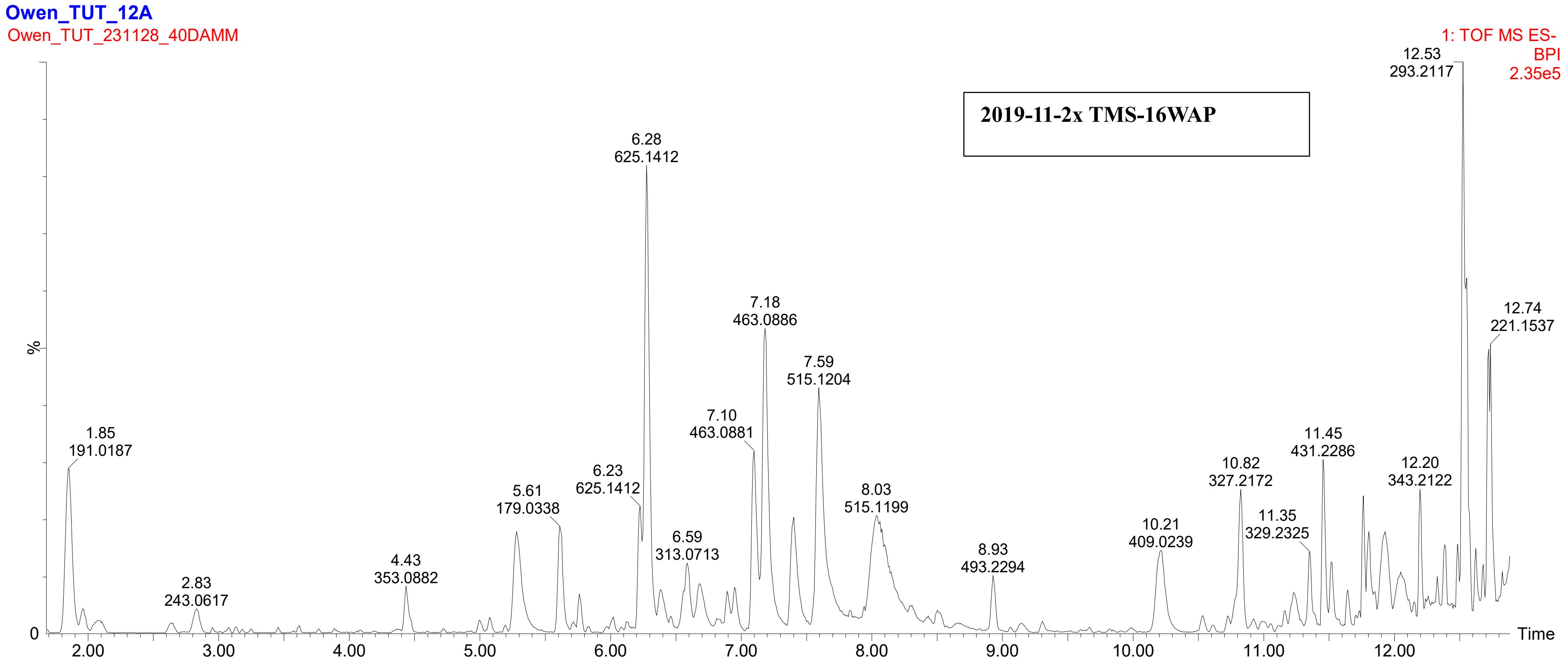

Owen_TUT_12A
Owen_TUT_231128_40DAMM
1: TOF MS ES-
BPI
2.35e5
2019-11-2x TMS-16WAP
%
1.85
191.0187
2.83
243.0617
4.43
353.0882
5.61
179.0338
6.23
625.1412
6.28
625.1412
6.59
313.0713
7.10
463.0881
7.18
463.0886
7.59
515.1204
8.03
515.1199
8.93
493.2294
10.21
409.0239
10.82
327.2172
11.35
329.2325
11.45
431.2286
12.20
343.2122
12.53
293.2117
12.74
221.1537
0
2.00
3.00
4.00
5.00
6.00
7.00
8.00
9.00
10.00
11.00
12.00
Time

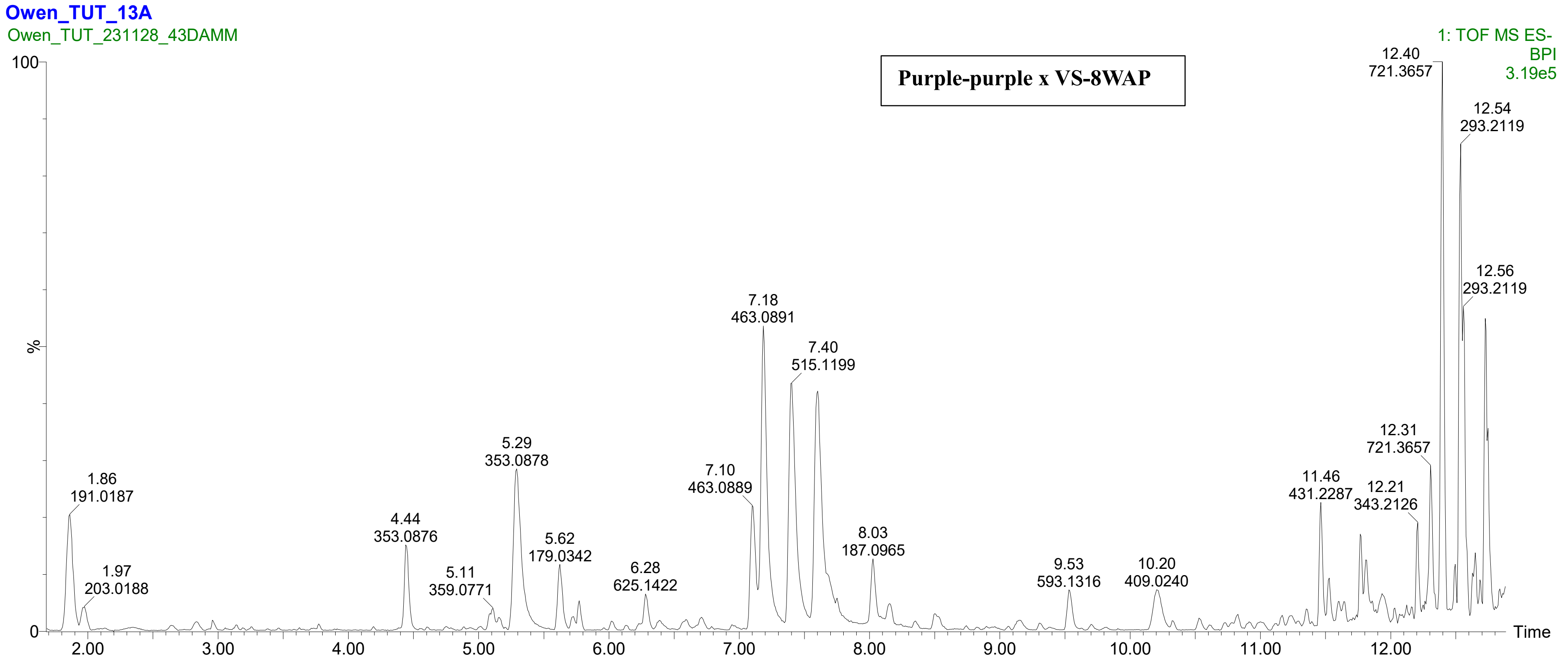

Owen_TUT_13A
Owen_TUT_231128_43DAMM
1: TOF MS ES-
BPI
3.19e5
Purple-purple x VS-8WAP
12.40
721.3657
12.54
293.2119
12.56
293.2119
7.18
463.0891
7.40
515.1199
12.31
721.3657
11.46
431.2287
12.21
343.2126
5.29
353.0878
1.86
191.0187
7.10
463.0889
4.44
353.0876
5.62
179.0342
8.03
187.0965
1.97
203.0188
5.11
359.0771
6.28
625.1422
9.53
593.1316
10.20
409.0240
100
%
0
2.00
3.00
4.00
5.00
6.00
7.00
8.00
9.00
10.00
11.00
12.00
Time

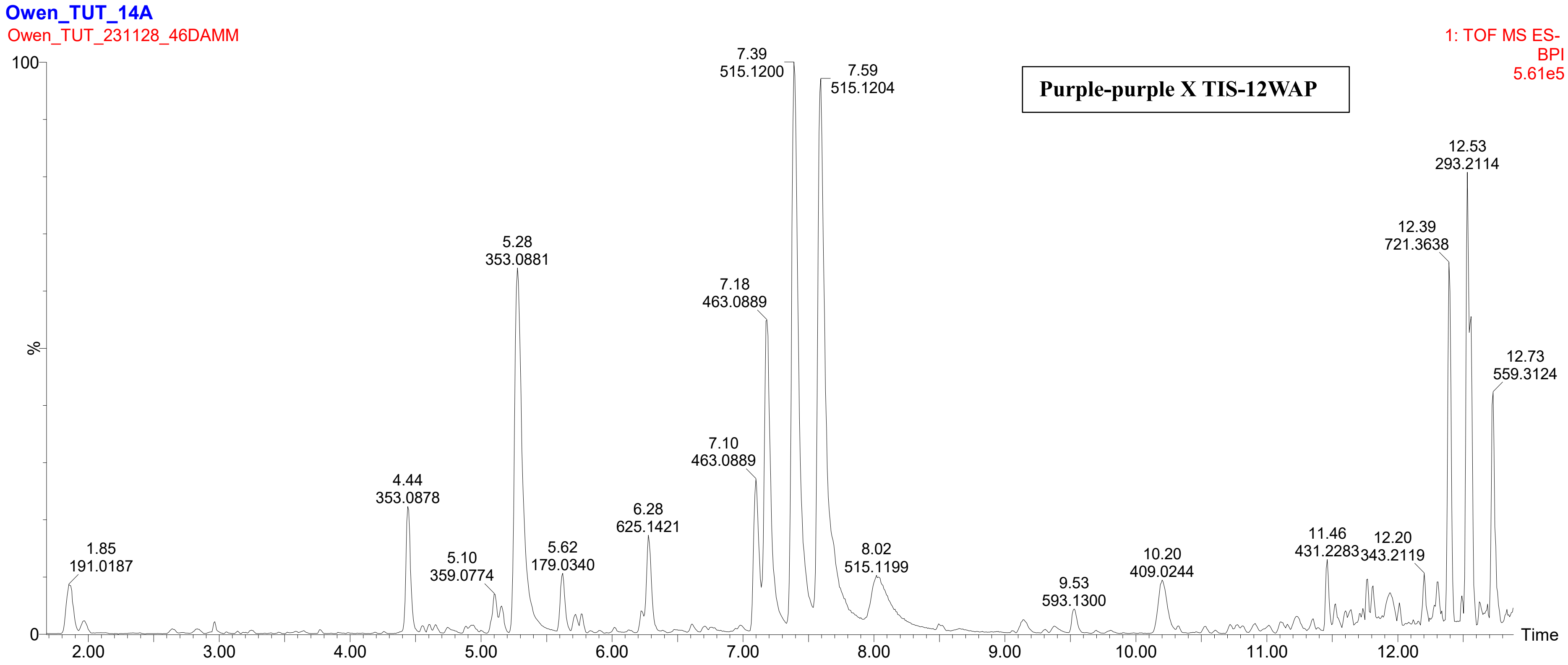
Owen_TUT_14A
Owen_TUT_231128_46DAMM
1: TOF MS ES-
BPI
5.61e5
Purple-purple X TIS-12WAP
100
%
0
7.39
515.1200
7.59
515.1204
12.53
293.2114
12.39
721.3638
5.28
353.0881
7.18
463.0889
12.73
559.3124
7.10
463.0889
4.44
353.0878
6.28
625.1421
1.85
191.0187
5.10
359.0774
5.62
179.0340
8.02
515.1199
11.46
431.2283
12.20
343.2119
10.20
409.0244
9.53
593.1300
2.00
3.00
4.00
5.00
6.00
7.00
8.00
9.00
10.00
11.00
12.00
Time

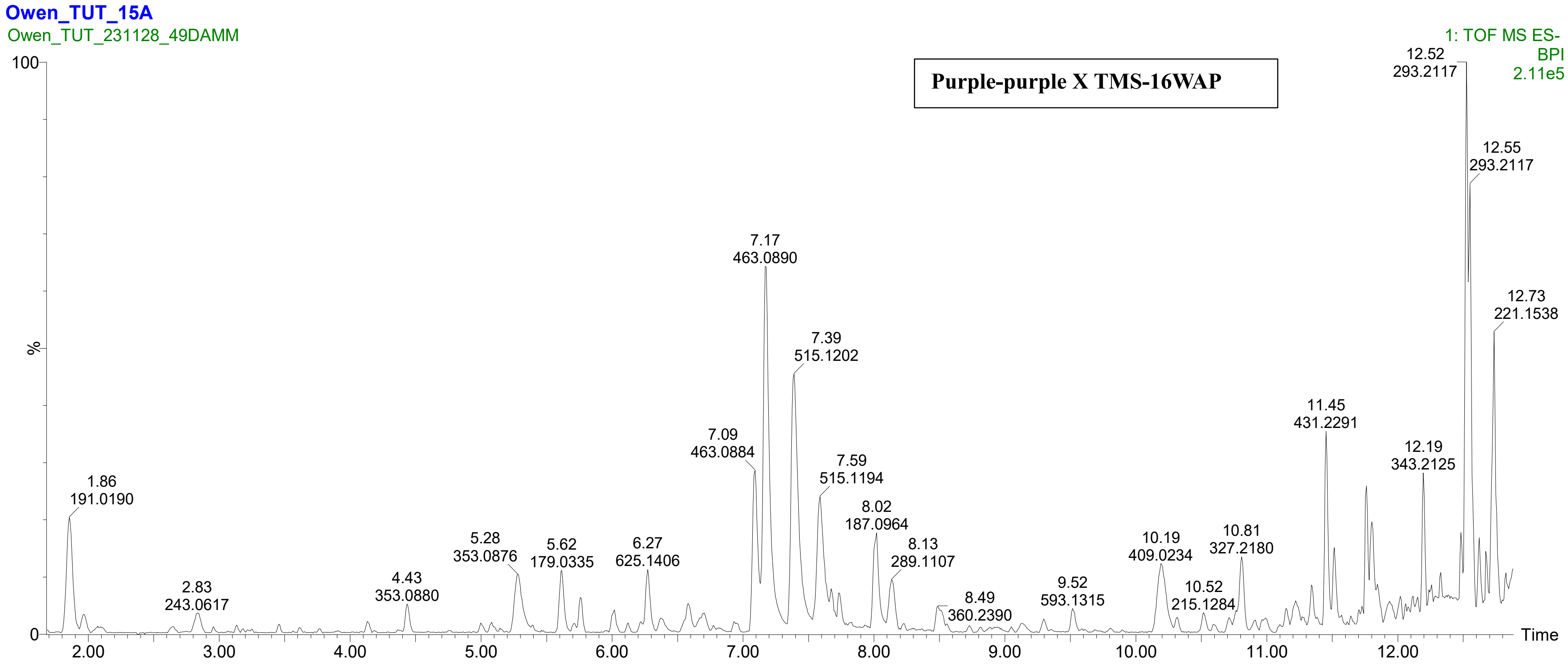

Supplementary Figure S4-S18. UPLC-QTOF/MS BPI chromatograms in ESI negative mode of different purple fleshed sweet potato genotypes at different stages of harvesting
